# Supplementary material for: Cell type‐specific regulation of ciliary transition zone assembly in vertebrates
Source: EMBO J. 2018 Apr 12;37(10):e97791. doi: 10.15252/embj.201797791 (PMC5978567; doi:10.15252/embj.201797791)
Supplement: Supplementary file 1 — Appendix [file EMBJ-37-e97791-s001.pdf]

Appendix for

Wiegering et al.,

“Cell type-specific regulation of ciliary transition zone assembly in vertebrates”

Appendix Contents

Appendix Materials and Methods

Appendix Figure S1

Appendix Figure S2

Appendix Figure S3

Appendix Figure S4

Appendix Figure S5

Appendix Figure S6

Appendix Figure S7

Appendix Figure S8

Appendix Figure S9

Appendix Figure S10

Appendix Figure S11

Appendix Figure S12

Appendix Figure S13

Appendix Table S1

Appendix Table S2

Appendix References

Appendix Materials and Methods

CRISPR/Cas9-mediated gene inactivation

SgRNAs were produced from vector pSpCas9(BB)-2A-Puro (PX459) V2.0, which also expresses Cas9 and a puromycin selectable marker. The PX459 vector was a gift from Feng Zhang (Addgene plasmid # 62988) (Ran et al, 2013). NIH3T3 and HEK293 cells were transfected using Lipofectamine 3000 (Invitrogen). 24 hours after transfection transient puromycin selection was performed for 36-48 hours to reduce the number of cells which did not take up the vector. Subsequently, after recovery of the surviving cell pool, single cells were cloned in 96-well plates. After 8 days wells were visually inspected for clonal growth, and from days 10-12 on apparent single clones were expanded.

For the inactivation of *RPGRIP1L* and *RPGRIP1* in HEK293 cells, DNA contigs for the relevant regions (*RPGRIP1L* exon 3, *RPGRIP1* exon 4) were assembled from raw sequencing data of irrelevant data sets (NCBI accession numbers: SRX316360-SRX316370).

For the inactivation of *Invs* (exon 4), *Cep290* (exon 10) and *Nphp1* (exon 1) in NIH3T3 cells, DNA contigs for the relevant regions were similarly assembled (NCBI accession numbers: SRX262827-SRX262830, SRX366528-572). Several cell line-specific nucleotide differences were detected in comparison to the reference genome for some of the contigs, as well as a single nucleotide polymorphism within the contig of *Invs* exon 4. These differences and all primer sets are listed in Source data for Appendix.

Amplified DNAs from 24-48 clones were analysed for genome editing by RFLP analysis using diagnostic restriction enzymes whose recognition sequences overlapped the Cas9 site in the target sequence. From the resulting set of clones (which appeared to have lost the diagnostic enzyme recognition sequence on all alleles) we chose 6-8 clones for cloning

individual alleles and subsequent DNA sequencing. The genotypes of all clones analysed are shown in Appendix Fig S3.

All clones analysed (n=38) are mutated on all alleles. In HEK293 derived clones (*RPGRIP1L* inactivation -6 clones, *RPGRIP1* inactivation -8 clones), we detected 2 alleles per clone, in NIH3T3 derived clones we either observed 3 alleles (*Invs*, *Nphp1*) or 4 alleles (*Cep290*). The latter is in accordance with karyotype analysis of the NIH3T3 cell line (Leibiger et al, 2013) which revealed that chromosome 4 (*Invs*) is present three times, chromosome 10 (*Cep290*) is present five times and chromosome 2 (*Nphp1*) is present four times in NIH3T3 cells. In a number of cases we found less than the expected number of alleles (Appendix Fig S3), suggesting that some alleles could not be recovered by amplification, probably due to a larger deletion reaching into the primer sequence(s). In addition, we could show that within a given clone set individual clones had independently gained an identical mutation (Appendix Fig S3). In the case of the *Invs* clone set, we were able to demonstrate that even within a given clone different alleles had independently gained an identical mutation. The presence of a single base pair allelic difference within the *Invs* contig allowed the discrimination between different alleles (Appendix Fig S3). We assume similar scenarios for the clone sets of *Cep290* and *Nphp1* on-targets, yet there is no additional allelic marker present in these contigs which allows discrimination between alleles carrying an identical mutation. From each of the five CRISPR/Cas9 mediated gene inactivations we chose 2-4 clones (15 clones alltogether) for further analysis. All these clones except two (*Cep290* 39-21, *Nphp1* 21-23) could be shown to be inactivated for the gene of interest, by initial inspection via immunostaining. In the case of clone 39-21, on one allele a large deletion (del-458, Appendix Fig S3) removes both splice sites of *Cep290* exon 10 (data not shown), and splicing between exon 9 (donor site) and exon 11 (acceptor site) would result in an in frame deletion of 90 bps. In the case of clone 21-23 we noticed that one of its alleles carries a large insertion (ins+224, Appendix Fig S3) with several

potential splice donor sites 5' prime to the position of the in frame stop codon (data not shown).

Off-target analysis

Potential off-target sites were retrieved using the CRISPR Design tool (Hsu et al, 2013) (crispr.mit.edu/) and the Cas-OFFinder tool (Bae et al, 2014) (www.rgenome.net/cas-offinder/). From the merged output tables a ranked list was constructed using the CRISPR Design tool. From this list the top 5-6 candidate sites were chosen for analysis (PAM type NRG), and in addition all off-target sites with 3 mismatches and a maximum of 1 mismatch in the PAM-proximal 10 positions (PAM type NGG). This set comprised 47 sites from which 4 sites were not analysed because the sites carried additional mismatches in the HEK293 or NIH3T3 genomes, and another 3 sites could not be analysed for technical reasons (see Source data for Appendix). The final set we analysed comprised 40 potential off-target sites (RPGRIP1L - 11 sites, RPGRIP1 - 5 sites, Invs - 8 sites, Cep290 - 9 sites, Nphp1 - 7 sites).

We characterised potential off-target mutations in the same clonal lines which we analysed for the targeting of the five on-target sites (6-8 clones per off-target set). We amplified all 40 off-target sites and tested for potential off-target mutations using RFLP analysis. Similar to the on-target analyses diagnostic restriction enzyme sites overlapping the Cas9 site were tested for their absence. Due to the individual sequence of a given off-target, a certain number of potential mutations of a particular type (mostly indels in the range of 1-3 bps) cannot be detected by the „leading" enzyme. These mutations often lead to the formation of novel enzyme recognition sites whose presence we tested by restriction with additional endonucleases (Appendix Figs S4-S8).

The RFLP analyses performed are summarised in Appendix Table S1. Since in a number of cases potential indel mutations (e.g. 3 bp insertions) could not be sufficiently detected by

RFLP analysis, we wanted to get an idea of which type of mutations occur most frequently in CRISPR/Cas9 targeted loci. We analysed a set of on-target mutations for the size distribution of indels (Appendix Fig S9). Deletions are observed in more than 70% of indels analysed, with small deletions (1-3 bps) occuring most frequently, and the frequency of larger deletions (4-20 bps) gradually decreasing. Insertions are observed in less than 30% of indels analysed, with the insertion of a single base pair occuring most frequently, but unlike deletions, a sharp drop in the frequency of larger insertions (2-20 bps) was observed. Insertions of 3 bps occur in less than 2% of all cases. In both categories, (very) large deletions and insertions (35-450 bps) are observed. We conclude that the limited detection of 3 bp-insertions is tolerable, rather than insufficient detection of both 1-3 bp deletions and 1 bp insertions in the same off-target (RPGRIP1L-OT2, Invs-OT4, Cep290-OT7, Nphp1-OT8, Nphp1-OT7). For this reason PCR products were cloned and sequenced for these off-targets (Appendix Table S2) and for Nphp1-OT2 (mutated in 3/7 clones by RFLP). All samples sequenced were unmutated, including Nphp1-OT2 from clones 21-21 and 21-23 which were also tested negative in the RFLP analysis.

In summary, we tested 40 off-target sites (4 OTs with 2 mismatches, 23 OTs with 3 mismatches, and 13 OTs with 4 mismatches) and only identified mutations in one off-target site for *Nphp1* (OT2 with 3 mismatches, 3/7 clones). We conclude that the off-target mutation rate is low for the sites we tested, similar to a previous study (Yang et al, 2013), where from 47 sites tested, mutations were only detected in 3 sites (2/2 OTs with 1 mismatch, 1/3 OTs with 2 mismatches, 0/35 OTs with 3 mismatches, 0/7 OTs with 4 mismatches).

The sequences of all off-targets analysed and additional information on their analysis is given in Source data for Appendix (page1), primer sets are listed in Source data for Appendix (page2).

Appendix Figures

Appendix Figure S1

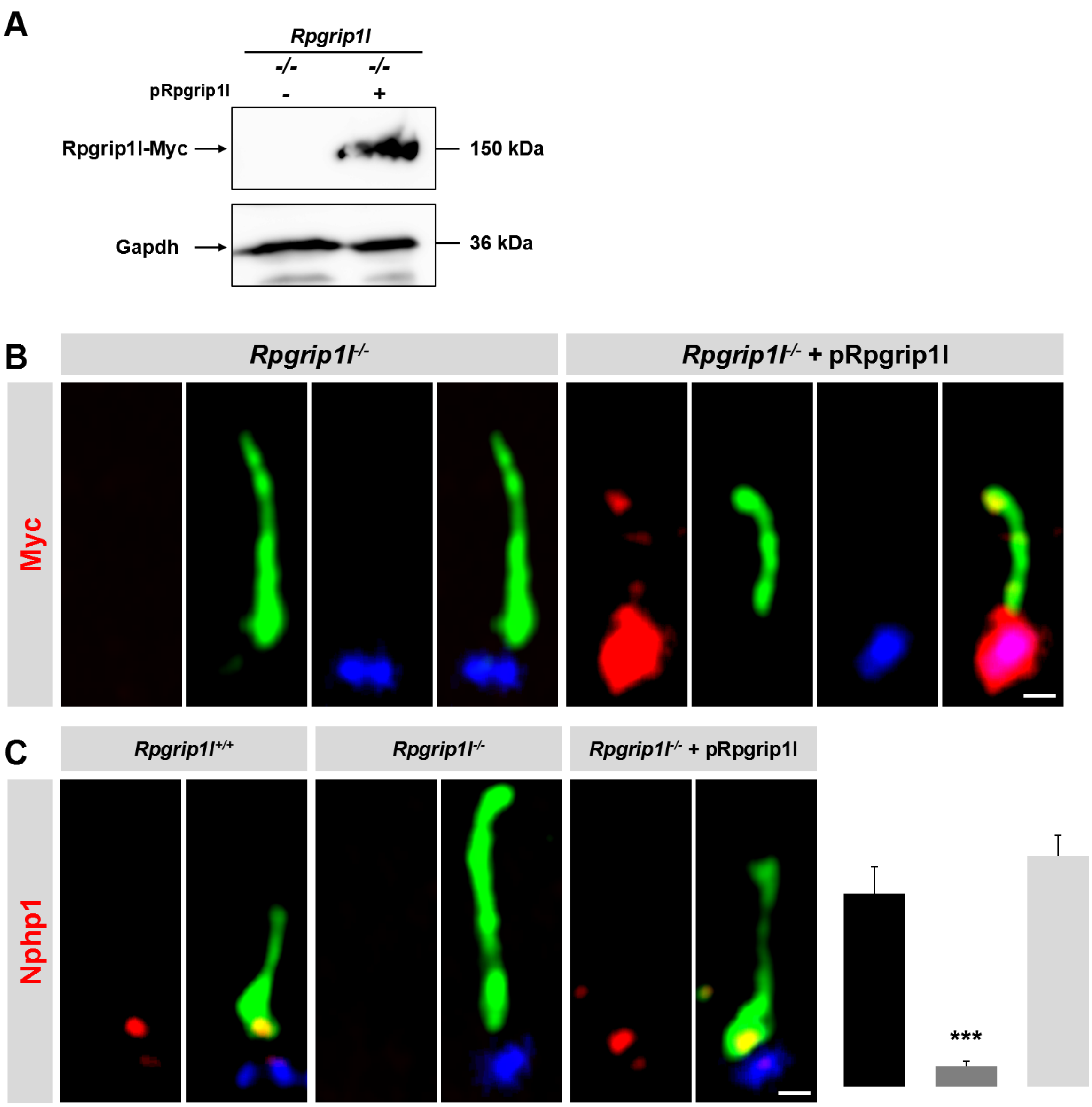

Appendix Figure S1. Rescue of the Nphp1 amount in *Rpgrip1l*$^{-/-}$ MEFs via Rpgrip1l re-expression. (A) Western blot analysis of cell lysates obtained from MEFs which were isolated from *Rpgrip1l*$^{-/-}$ (n=3) embryos. Cells were transfected with a plasmid encoding human full-length Rpgrip1l with a Myc-Tag (pRpgrip1l). Gapdh serves as loading control. (B) Immunofluorescence on *Rpgrip1l*$^{-/-}$ and transfected *Rpgrip1l*$^{-/-}$ MEFs. Scale bar (in white) represents a length of 0.5 µm. The ciliary axoneme is stained in green by acetylated α-tubulin

and the BB in blue by γ-tubulin. Myc is shown in red. (C) Immunofluorescence on WT, *Rpgrip1l*$^{-/-}$ and transfected *Rpgrip1l*$^{-/-}$ MEFs. Scale bar (in white) represents a length of 0.5 µm. The ciliary axoneme is stained in green by detyrosinated α-tubulin and the BB in blue by γ-tubulin. Nphp1 is shown in red. At least, 20 cilia per individual were used for quantification. The black bar represents the quantification in WT MEFs, the dark grey bar the quantification in *Rpgrip1l*$^{-/-}$ MEFs and the bright grey bar the quantification in transfected *Rpgrip1l*$^{-/-}$ MEFs. The WT bar was normalised to 100%. Statistical Data are shown as mean ± s.e.m. Asterisks denote statistical significance according to one-way ANOVA and Tukey HSD tests (*** $P<0.001$) ($F(2,56)=24,97$, $P<0.0001$).

Appendix Figure S2

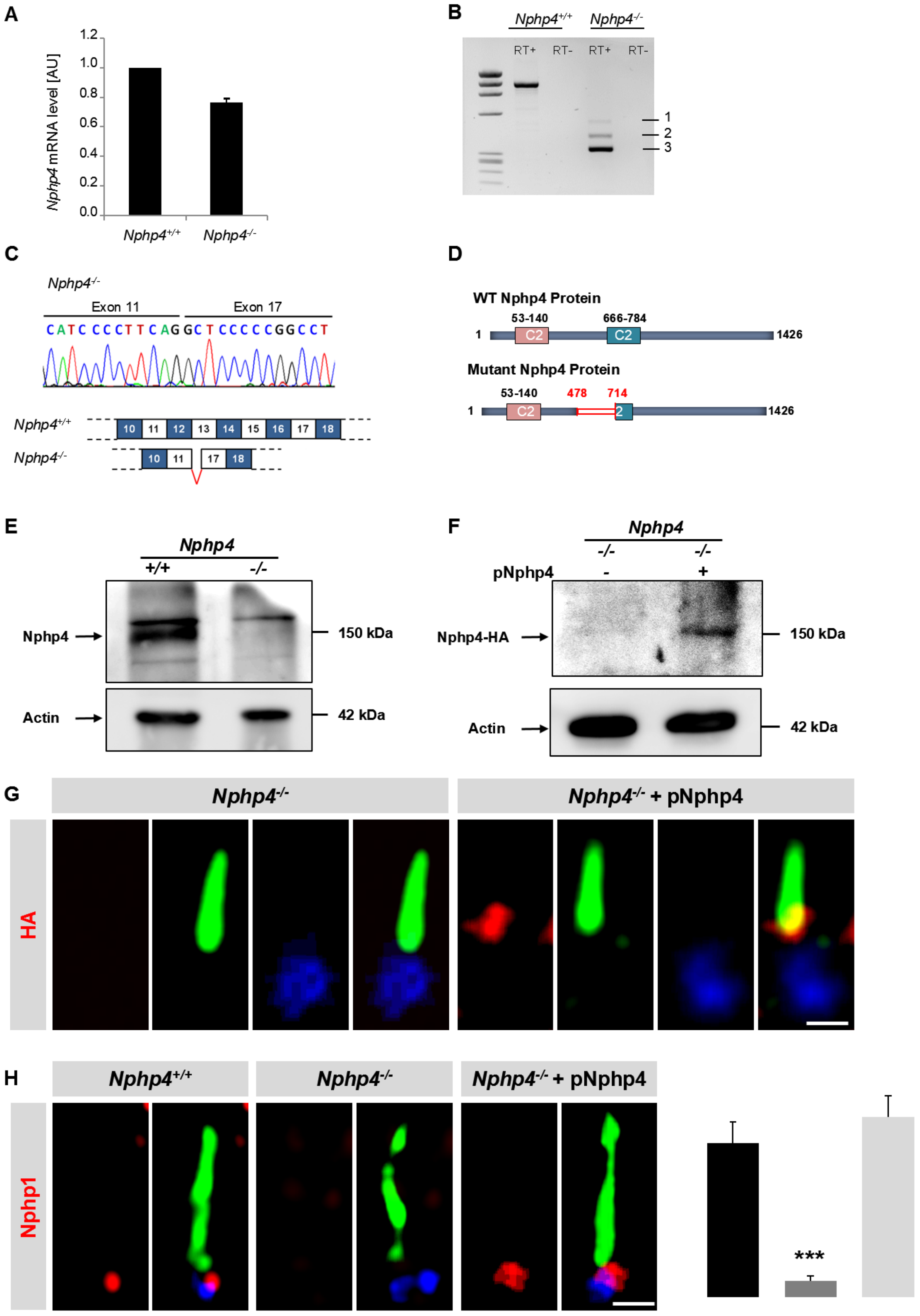

Appendix Figure S2. Characterisation of the *Nphp4* mutation which was introduced in mice. (A) Real-time qRT-PCR reveals a decrease of 30% of *Nphp4* mRNA expression level in *Nphp4*$^{-/-}$ compared to *Nphp4*$^{+/+}$ MEFs. AU, arbitrary unit. (B) RT-PCR amplification of the cDNA region between exon 10 - exon 17 shows 3 shorter transcripts in *Nphp4*$^{-/-}$ compared to WT MEFs: two out-of-frame transcripts (1: deletion of exon 14 - exon 16; 2: deletion of exon 13 - exon 16) and one in-frame transcript (3: deletion of exon 12 - exon 16). (C) Chromatograms from Sanger sequencing of PCR products from *Nphp4*$^{-/-}$ MEFs obtained as in (B) and schematic representation showing the main abnormal transcript lacking exon 12 - exon 16. (D) Schematic representation of the predicted abnormal Nphp4 protein lacking AA478 to 714 encompassing part of the C2 domain (AA 666-784) known to interact with RPGRIP1, RPGRIP1L (Arts et al, 2007) and KIF13B (Schou et al, 2017). (E) Western blot studies elucidate the Nphp4 protein is absent in MEFs which were obtained from *Nphp4*$^{-/-}$ mouse embryos. (F) Western blot analysis of lysates obtained from *Nphp4*$^{-/-}$ MEFs and from *Nphp4*$^{-/-}$ MEFs transfected with a plasmid encoding a Nphp4 (full-length)-HA fusion protein. (E and F) Actin serves as loading control. (G) Immunofluorescence on *Nphp4*$^{-/-}$ MEFs and on *Nphp4*$^{-/-}$ MEFs transfected with a plasmid encoding a Nphp4 (full-length)-HA fusion protein (in red). The ciliary axoneme is stained in green by acetylated α-tubulin and the BB in blue by γ-tubulin. The scale bar (in white) represents a length of 1 μm. (H) Immunofluorescence on WT MEFs, on *Nphp4*$^{-/-}$ MEFs and on *Nphp4*$^{-/-}$ MEFs transfected with a plasmid encoding a Nphp4 (full-length)-HA fusion protein. The ciliary axoneme is stained in green by detyrosinated α-tubulin and the BB in blue by γ-tubulin. The scale bar (in white) represents a length of 1 μm. Nphp1 (in red) was quantified. At least, 20 cilia per individual were used for quantification. The black bar represents the normalised quantification in WT MEFs, the dark grey bar the quantification in *Nphp4*$^{-/-}$ MEFs and the bright grey bar the quantification in *Nphp4*$^{-/-}$ MEFs which were transfected with a plasmid encoding a Nphp4 (full-length)-HA

fusion protein. Data are shown as mean ± s.e.m. Asterisks denote statistical significance according to one-way ANOVA and Tukey HSD tests (* $P<0.05$; ** $P<0.01$; *** $P<0.001$) ($F(2,60)=21,74$, $P<0,0001$).

Appendix Figure S3

A **RPGRIP1L exon3** (HEK293)

```
                                                                    ****            ****  ***    ****
                         CCAnnnnnnTGG  BstXI                        1-14  1-16  1-26  1-28  1-7   1-23
AGCGGGTTGGTGGCGGCCCCAAGCGGCTGGGACGAGATGTGGAAATGGAAGA      WT
AGCGGGTTGGTGGCGGC-----GCGGCTGGGACGAGATGTGGAAATGGAAGA      del-5                               1-7
AGCGGGTTGGTG----------GCGGCTGGGACGAGATGTGGAAATGGAAGA      del-10          1-16        1-28
AGCGGGTCG------------AGCGGCTGGGACGAGATGTGGAAATGGAAGA      del-12                1-26
AGCGGGTTG-------------GCGGCTGGGACGAGATGTGGAAATGGAAGA      del-13                      1-28
AGCGGGTTGGT-------------GGCTGGGACGAGATGTGGAAATGGAAGA      del-13                                    1-23
AGCGGGTTGGTGGCGGCCCCAAGGCGGCTGGGACGAGATGTGGAAATGGAAGA     ins+G     1-14        1-26
AGCGGGTTGGTGGCGGCCCCAAGGGCGGCTGGGACGAGATGTGGAAATGGAAGA    ins+2     1-14
nnnnnnnnnnnnnnnnnnnnnnGCGGCTGGGACGAGATGTGGAAATGGAAGA      ins+45          1-16
                         number of different alleles          2     2     2     2     1     1
                         alleles out of frame                 2/2   1/2   1/2   2/2   1/1   1/1
```

B **RPGRIP1 exon4** (HEK293)

```
                                                                              ****        ****  ****
                        CCATC  BccI                               3-3   3-6   3-25  3-29  3-30  3-31  3-34  3-11
GCTGAGCTACACAGCCCCTCCATCGTTTAAGGAGCATGCGACAAATGAAAAC      WT
GCTGAGCTACACAGCCCCTCCAT-GTTTAAGGAGCATGCGACAAATGAAAAC      del-1                 3-25
GCTGAGCTACACAGCCCCTCC---GTTTAAGGAGCATGCGACAAATGAAAAC      del-3     3-3
GCTGAGCTACACAGC-------TCGTTTAAGGAGCATGCGACAAATGAAAAC      del-7                       3-29
GCTGAGCTACACAGCCCCTCCAT--------GAGCATGCGACAAATGAAAAC      del-8                                   3-31
GCTGAGCTACACAGCCCCTCCA-----------GCATGCGACAAATGAAAAC      del-11    3-3
GCTGAGCTACACAGCCCCTCCA------------CATGCGACAAATGAAAAC      del-12          3-6
GCTGAGCTACACAGCCCCTCCA--------------TGCGACAAATGAAAAC      del-14                                  3-31  3-34
GCTGANCTACACAGCC------------------------------------      del-92                      3-29
----------------------------------------------------      del-232                           3-30
GCTGAGCTACACAGCCCCTCCATTCGTTTAAGGAGCATGCGACAAATGAAAAC     ins+T                                         3-34  3-11
GCTGAGCTACACAGCCCCTCCAATCGTTTAAGGAGCATGCGACAAATGAAAAC     ins+A                             3-30
GCTGAGCTACACAGCCCCTCCACATCGTTTAAGGAGCATGCGACAAATGAAAAC    ins+2                 3-25
nnnnnnnnnnnnnnnnnnnnnnTCGTTTAAGGAGCATGCGACAAATGAAAAC      ins+253         3-6
                         number of different alleles          2     2     2     2     2     2     2     1
                         alleles out of frame                 1/2   1/2   2/2   2/2   2/2   2/2   2/2   1/1
```

C **Invs exon4** (NIH3T3)

```
                                                                        ****                        *****
                        GACGTG  BmgBI                             48-5  48-7  48-18  48-10  48-17  48-20  48-28  48-31
CTCCAACATCGGGATTCCCGACGTGGAAGGCAAGATCCCACTCCACTGGGCA      WT
CTCCAACATCGGGATTCCCG--GTGGAAGGCAAGATCCCACTCCACTGGGCA      del-2^T   48-5
CTCCAACATCGGGATTCCCGAC--GGAAGGCAAGATCCCACTCCACTGGGCA      del-2^A         48-7
CTCCAACATCGGGATTCC----GTGGAAGGCAAGATCCCACTCCACTGGGCA      del-4^A                                                48-31
CTCCAACATCGGGA--------GTGGAAGGCAAGATCCCACTCCACTGGGCA      del-8^A               48-18
CTCCAACATCGGGA-------------AGGCAAGATCCCACTCCACTGGGCA      del-13^A              48-18
CTCCAACATCGGGA--------------GGCAAGATCCCACTCCACTGGGCA      del-14^A                                   48-20
CTCCAACATCGGGAT--------------------TCCCACTCCACTGGGCA      del-20^T              48-18
CTCCA---------------------------------------ACTGGGCA      del-39^A  48-5
CTCCA----------------------------------------CTGGGCA      del-40^T                     48-10
CTCCA----------------------------------------CTGGGCA      del-40^A                     48-10
CTCCAACATCGGGATTCCCGACGGTGGAAGGCAAGATCCCACTCCACTGGGCA     ins+G^T         48-7                48-17         48-28
CTCCAACATCGGGATTCCCGACGGTGGAAGGCAAGATCCCACTCCACTGGGCA     ins+G^A         48-7                48-17  48-20  48-28
nnnnnnnnnnnnnnnnnnnnnnGTGGAAGGCAAGATCCCACTCCACTGGGCA      ins>250   48-5
                         number of different alleles          3     3     3      2      2      2      2      1
                         alleles out off frame                (1/3) 3/3   3/3    2/2    2/2    2/2    2/2    1/1
```

D **Cep290 exon10** (NIH3T3)

```
                                                                        *****        *****  *****         *****
                         TCATGA  BspHI                            39-12 39-51 39-6   39-21  39-53  39-9   39-10  39-34
GGATGAAGAGGACGATCCAGTCATGATGGCTGTTAACGCAAAAGTGGAAGAG      WT
GGATGAAGAGGACGATCCAGTCGTGATGGCTGTTAACGCAAAAGTGGAAGAG      A to G                39-6
GGATGAAGAGGACGATCCAGT-ATGATGGCTGTTAACGCAAAAGTGGAAGAG      del-C     39-12 39-51
GGATGAAGAGGACGATCCAGTCA-GATGGCTGTTAACGCAAAAGTGGAAGAG      del-T                        39-21
GGATGAAGAGGACGATCCAGTCA----GGCTGTTAACGCAAAAGTGGAAGAG      del-4                                                    39-34
GGATGAAGAGGACGATCCAGTC--------TGTTAACGCAAAAGTGGAAGAG      del-8           39-51               39-53
GGATGAAGAGGACGATCCAGTCA----------TAACGCAAAAGTGGAAGAG      del-10                                     39-9
GGATGTAGAGGA-----------------CTGTTAACGCAAAAGTGGAAGAG      del-17          39-51
GGATGAAGAGGA---------------GGCTGTTAACGCAAAAGTGGAAGAG      -15/+8    39-12
GGAT------------------------------------------GAAGAG      del-42                                     39-9
----------------------------------------------GAAGAG      del-226                                           39-10
----------------------ATGATGGCTGTTAACGCAAAAGTGGAAGAG      del-350               39-6
----------------------------------------------------      del-458                      39-21
GGATGAAGAGGACGATCCAGTCAATGATGGCTGTTAACGCAAAAGTGGAAGAG     ins+A     39-12 39-51 39-6   39-21  39-53         39-10
GGATGAAGAGGACGATCCAGTCATATGATGGCTGTTAACGCAAAAGTGGAAGAG    ins+2                               39-53
nnnnnnnnnnnnnnnnnnnnnnATGATGGCTGTTAACGCAAAAGTGGAAGAG      ins+205   39-12
nnnnnnnnnnnnnnnnnnnnnnATGATGGCTGTTAACGCAAAAGTGGAAGAG      ins+440                                                  39-34
                         number of different alleles          4     4     3      3      3      2      2      2
                         alleles out of frame                 4/4   4/4   2/3    3/3    3/3    1/2    2/2    2/2
```

E **Nphp1 exon1** (NIH3T3)

```
                                                                  21-2  *****               *****
                               CCGCGG  SacII                      21-18 21-21 21-11 21-13 21-23 21-27 21-29
GACCCGCTGCAGGCGCTGAGGCGCCGCGGGCAGGAGCTGAAGCTGCAGGTAG      WT
GACCCGCTGCAGGCGCTGAGGCGC-GCGGGCAGGAGCTGAAGCTGCAGGTAG      del-C     21-18 21-21                   21-27 21-29
GACCCGCTGCAGGCGCTGAGGCGCC-CGGGCAGGAGCTGAAGCTGCAGGTAG      del-G     21-18
GACCCGCTGCAGGCGCTGAGGCGC--CGGGCAGGAGCTGAAGCTGCAGGTAG      del-2                             21-23
GACCCGCTGCAGGCGCTGAGGCGC---GGGCAGGAGCTGAAGCTGCAGGTAG      del-3     21-18       21-11 21-13             21-29
GACCCGCTGCAGGCGCTGAGGCGC------CAGGAGCTGAAGCTGCAGGTAG      del-6                                   21-27
GACCCGCTGCAGGCGCTGAGG--------GCAGGAGCTGAAGCTGCAGGTAG      del-8           21-21
GACCCGCTGCAGGCGCTGAGGCGCGCGCGGGCAGGAGCTGAAGCTGCAGGTAG     ins+G                       21-13
GACCCGCTGCAGGCGCTGAGGCGCACGCGGGCAGGAGCTGAAGCTGCAGGTAG     ins+A           21-21
GACCCGCTGCAGGCGCTGAGCGCGCGGCGCGGGCAGGAGCTGAAGCTGCAGGTAG   ins+3                 21-11
nnnnnnnnnnnnnnnnnnnnnnnnCGCGGGCAGGAGCTGAAGCTGCAGGTAG      ins+224                           21-23
                         number of different alleles          3     3     2     2     2     2     2
                         alleles out of frame                 2/3   3/3   0/2   1/2   2/2   1/2   1/2
```

Appendix Figure S3. **Genotype analysis of targeted on-target alleles.**

(A-E) Sequences of targeted alleles are compared to the sequence of WT loci (on the left side), analysed clones and their genotype are indicated on the right side of the figure. For all genes targeted the coding strand is shown, the positions of PAM sequences are coloured in cyan, the positions of the 20 bp target sequences are coloured in yellow. Note that the authentic target and PAM sequences are located on the opposite strand for all targets shown, except for *Nphp1*-exon1 (authentic target sequences are available in Source data for Appendix). Deletions are indicated by dashes, small insertions of a few base pairs are shown in boldface, larger insertions are depicted as runs of „n“. The recognition sequences for the diagnostic restriction enzymes used in RFLP analyses to discriminate between mutated and WT loci are displayed above the WT sequence. (A) Genotype analysis of HEK293 clones targeted for *RPGRIP1L*. (B) Genotype analysis of HEK293 clones targeted for *RPGRIP1* (double inactivation of *RPGRIP1L* and *RPGRIP1*). A mixture of cells from 4 clones targeted for *RPGRIP1L* (clones 1-7, 1-14, 1-23, 1-28) was subsequently inactivated for *RPGRIP1*. Clone 3-30 is derived from clone 1-14, clone 3-31 is derived from clone 1-7, and clone 3-25 is derived from either clones 1-23 or 1-28 (re-genotyped by RFLP analysis, data not shown). In all HEK293 derived clones we observed at most 2 mutated alleles. (C) Genotype analysis of NIH3T3 clones targeted for *Invs*. The *Invs* exon4 locus was found to be present three times in the NIH3T3 cell line. Alleles can be distinguished by a single nucleotide polymorphism (A/T) 94 bps 3’ prime to the location of the Cas9 site in the intron between exon 4 and exon 5 (data not shown). A number of targeted clones (e.g. 48-7, 48-17 and 48-28) were identified which aquired an identical mutation (ins+G), both in independent clones and within a clone on different alleles (distinguishable by the allelic marker). The only clones in the collection of *Invs* targeted clones which appear to have only 2 amplifiable loci are clones 48-20 and 48-31 (T-allele is missing, data not shown), presumably due to a larger deletion reaching into the

primer sequence(s). One allele of clone 48-5 (ins>250) has not been sequenced. (D) Genotype analysis of NIH3T3 clones targeted for *Cep290*. The *Cep290* exon 10 locus was found to be present four times in clones 39-12 and 39-51. For all other clones we observed either 3 alleles (3x) or 2 alleles (3x) which are distinguishable by sequence. Note that in this clone set a number of identical mutations (ins+A (6x), del-C (2x) and del-8 (2x)) occurred independently in different clones, yet an allelic marker to distinguish these alleles from each other does not exist. (E) Genotype analysis of NIH3T3 clones targeted for *Nphp1*.The *Nphp1* exon 1 locus was found to be present 3 times in clones 21-2, 21-18 and 21-21. For all other clones we observed 2 alleles which are distinguishable by sequence. Note that in this clone set again a number of identical mutations (del-C (4x) and del-3 (4x)) occurred independently in different clones. Clones 21-2 and 21-18 turned out to be sister clones. (A-E) Clones used in the present study are marked by asterics (*) in cyan. Two other clones (*Cep290* - 39-21 and *Nphp1* - 21-23), although carrying only out of frame alleles, were tested positive by immunostaining, presumably due to alternative/abberant splicing (for details see Appendix Materials and Methods).

Appendix Figure S4

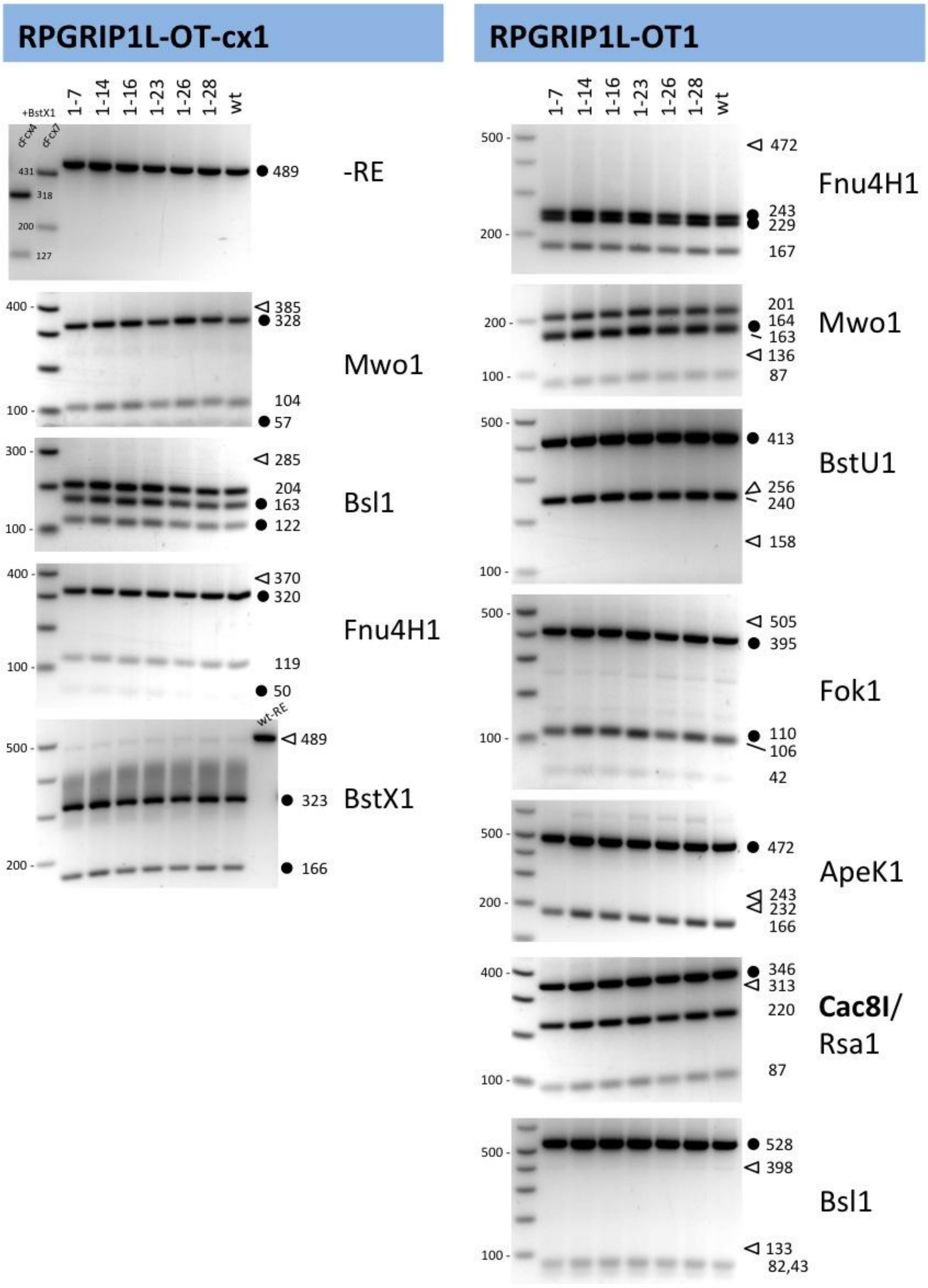

Appendix Figure S4 continued

RPGRIP1L-OT1

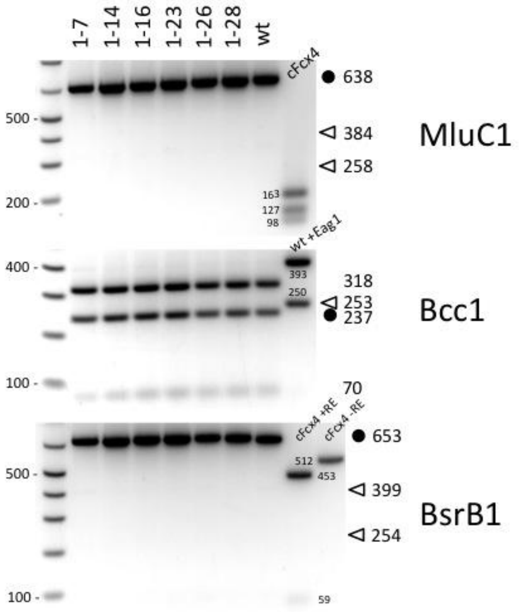

RPGRIP1L-OT-cx2

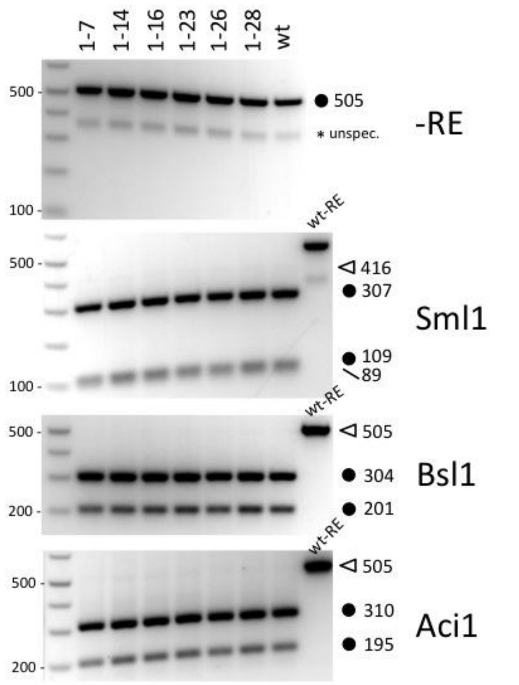

RPGRIP1L-OT2

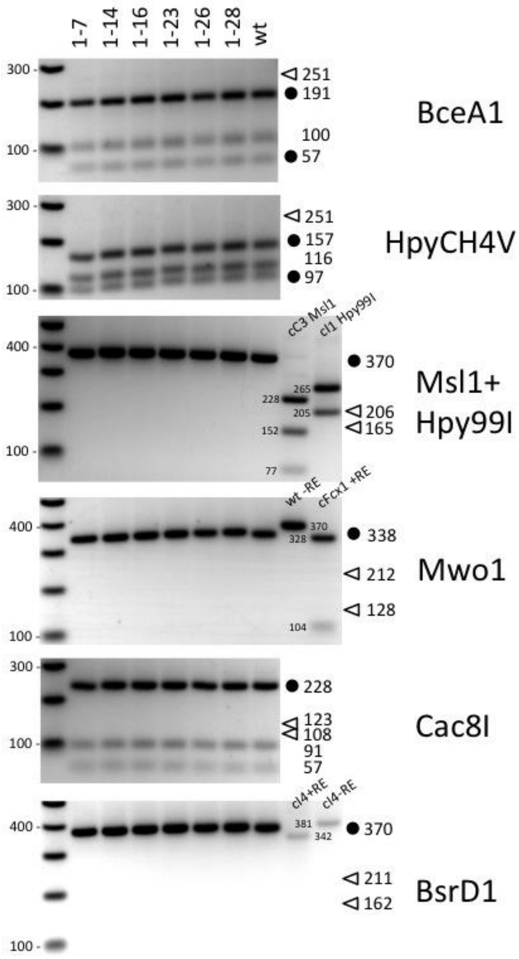

Appendix Figure S4 continued

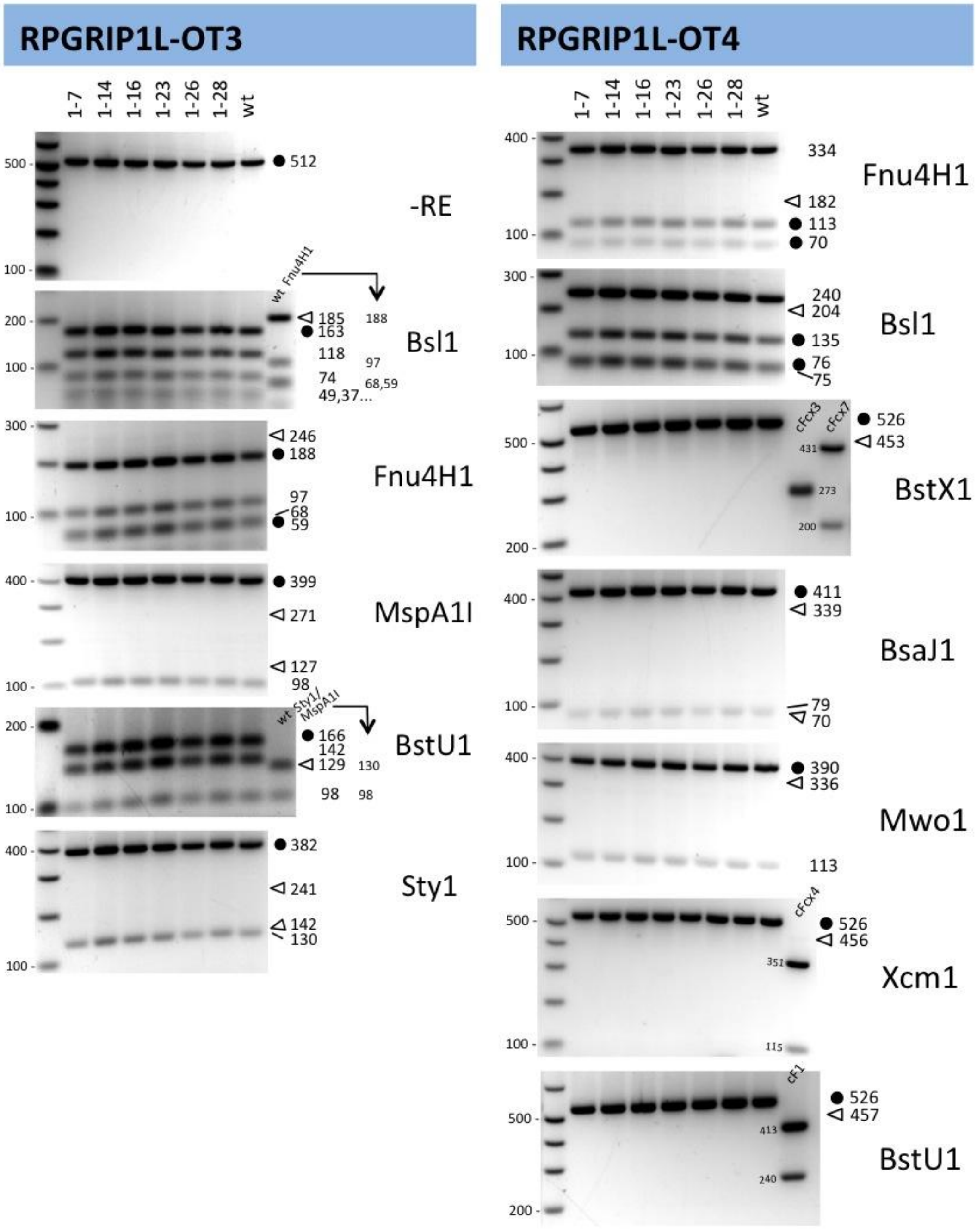

Appendix Figure S4 continued

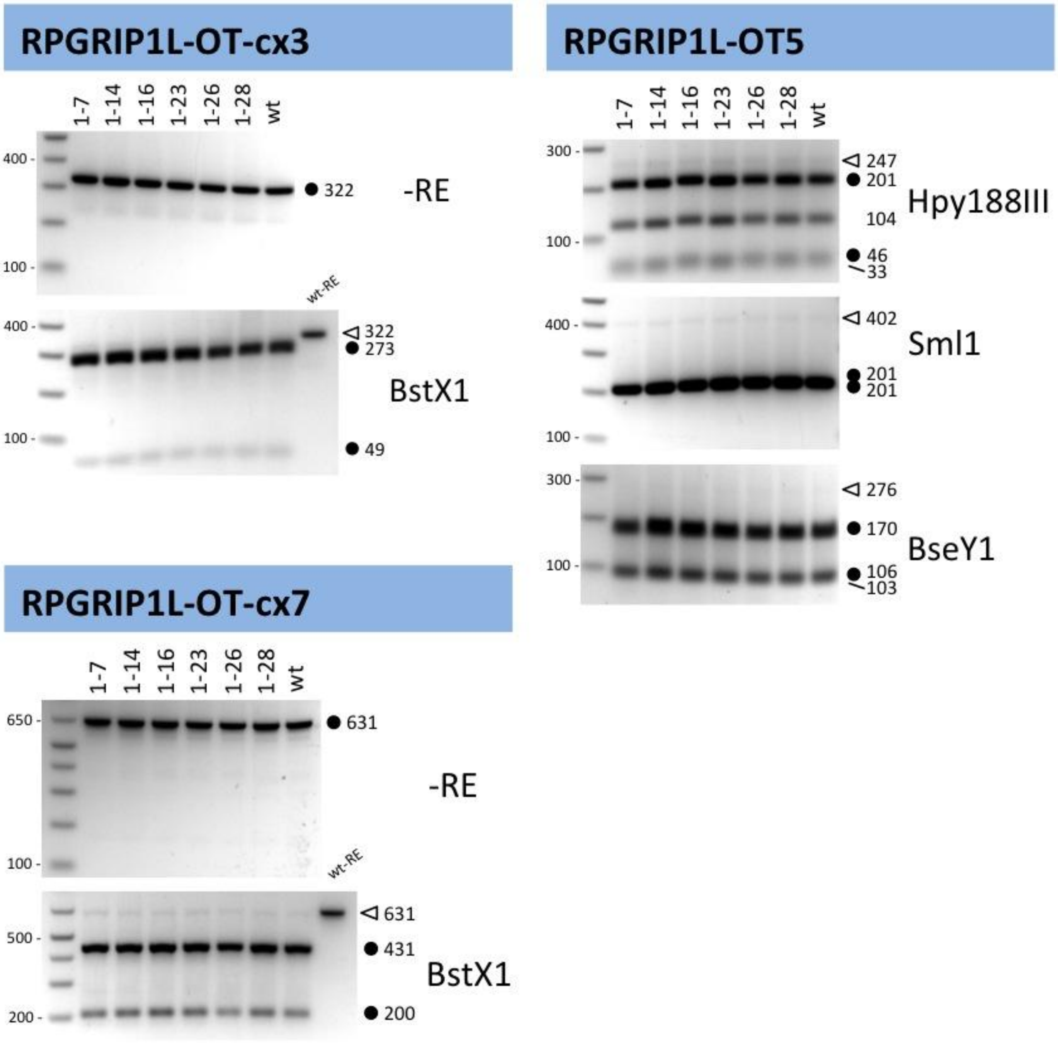

Appendix Figure S4 continued

RPGRIP1L-OT-cx4

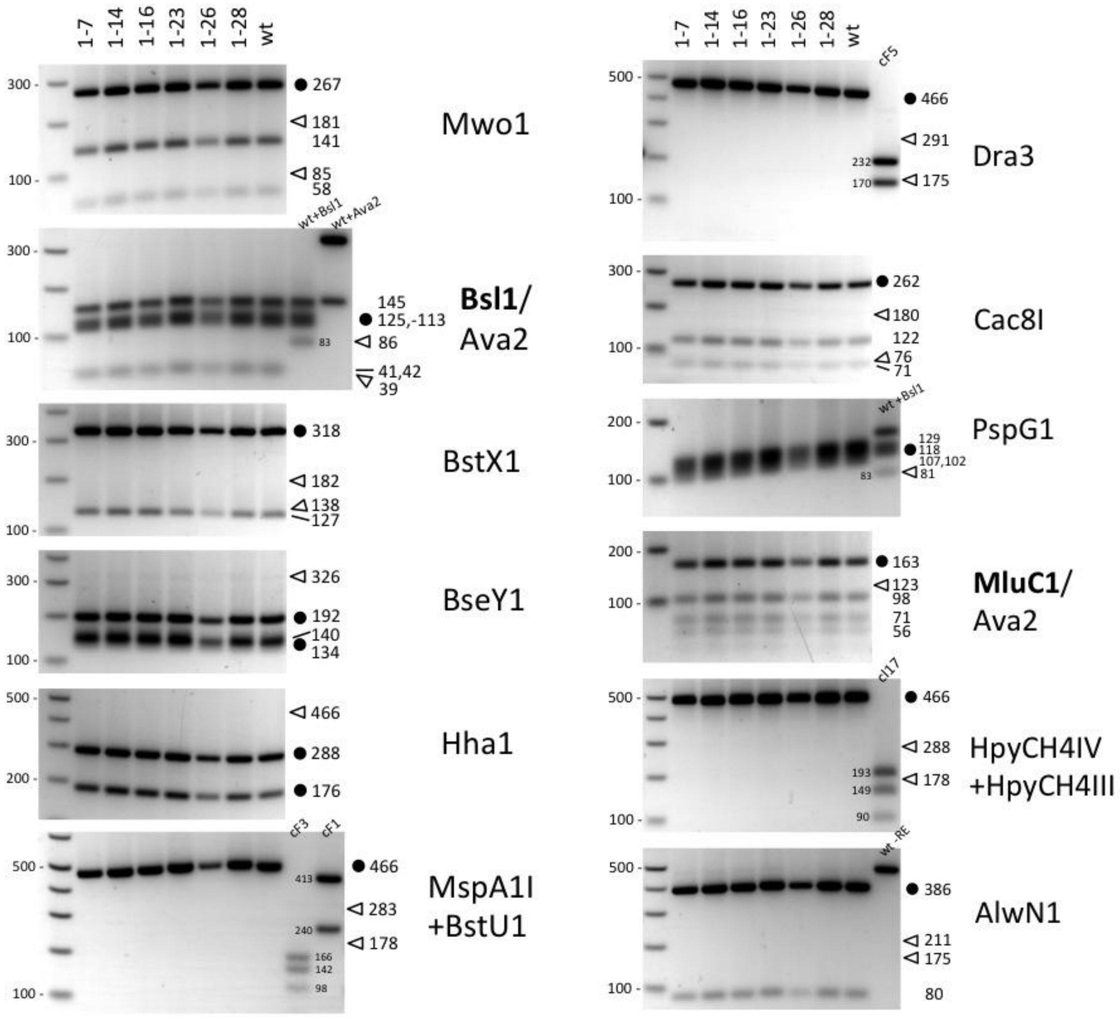

Appendix Figure S4 continued

RPGRIP1L-OT-cx6

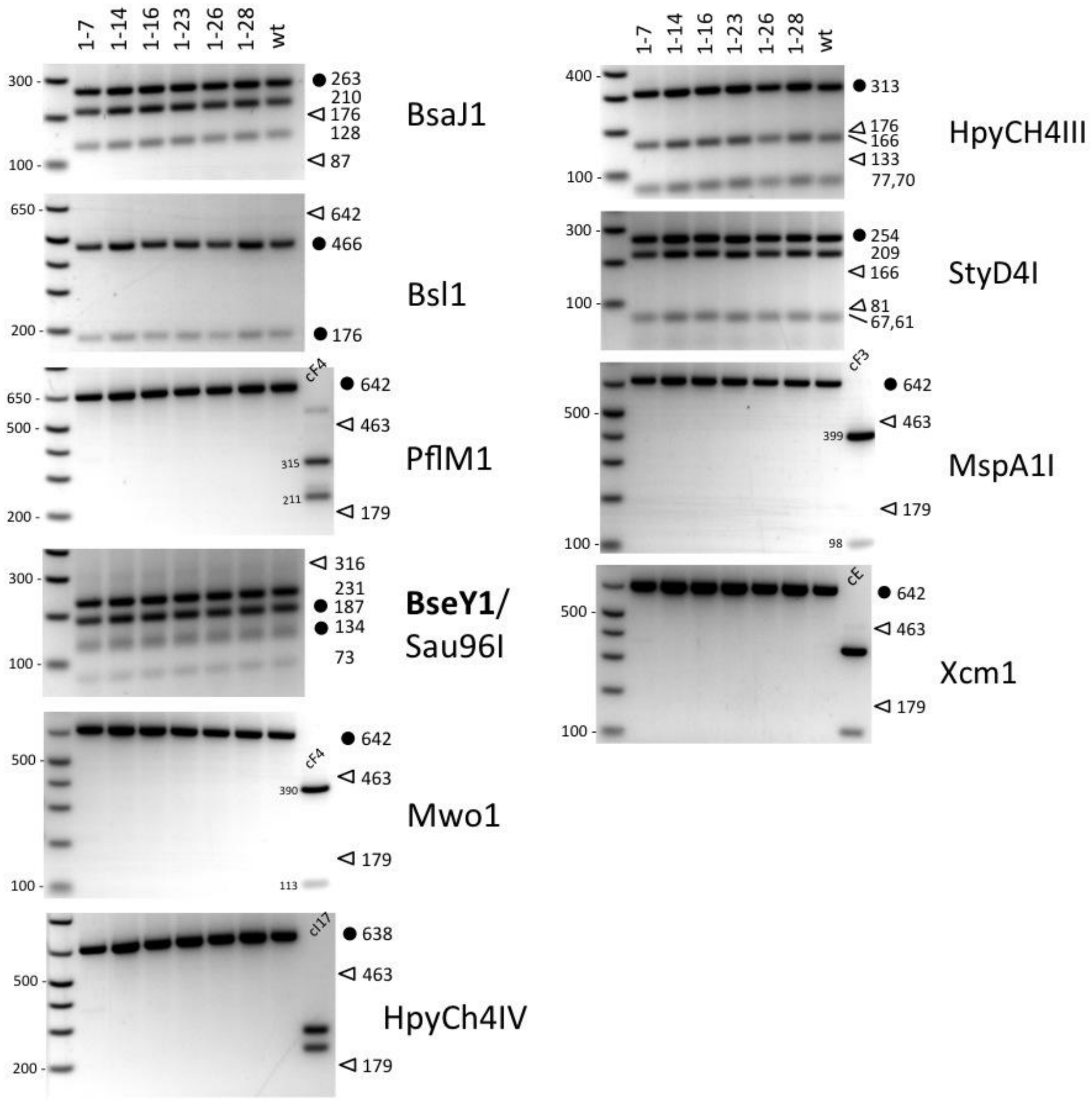

Appendix Figure S5

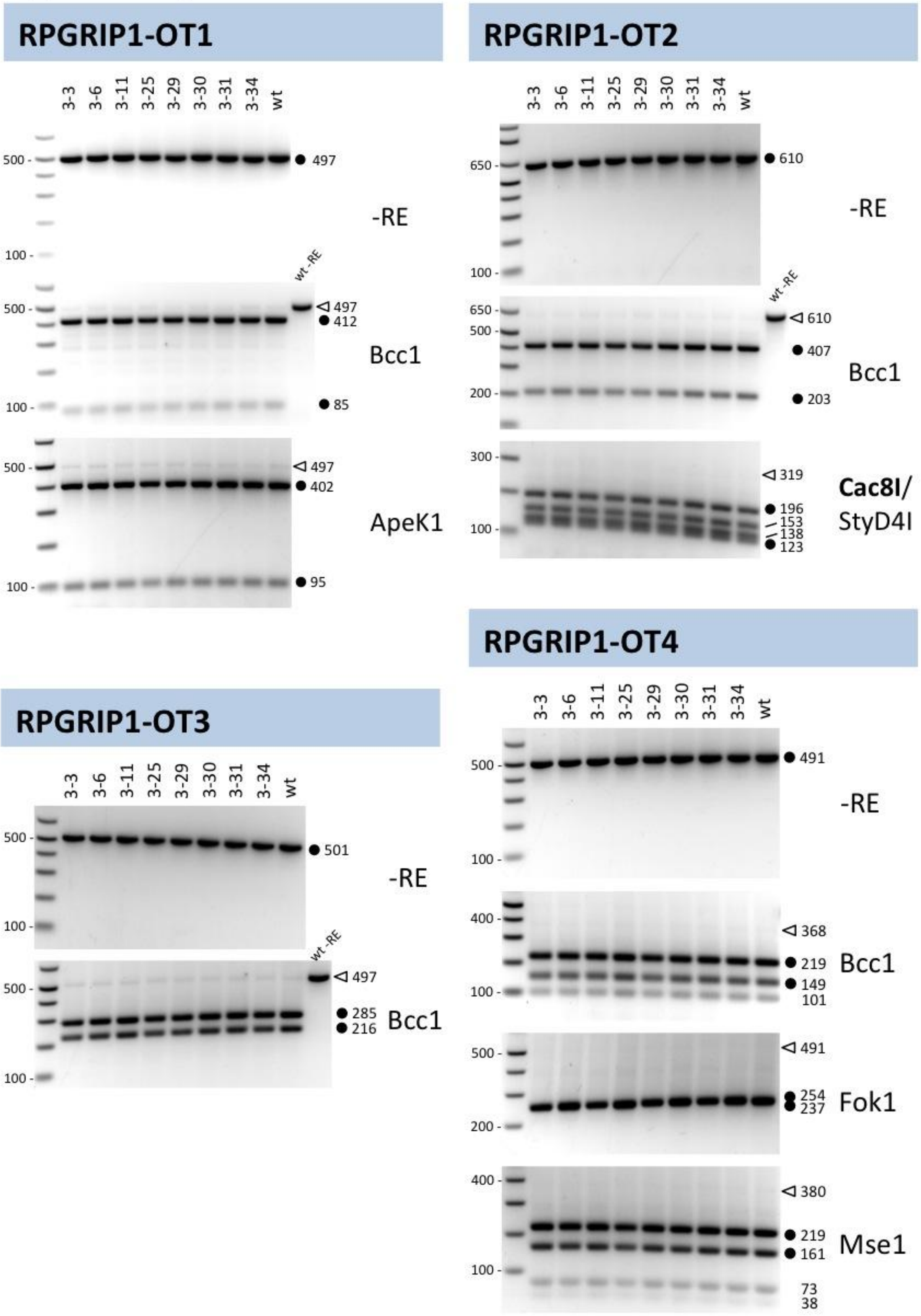

Appendix Figure S5 continued

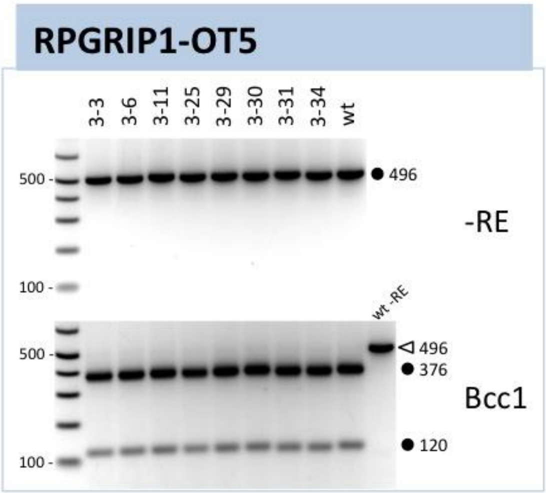

Appendix Figure S6

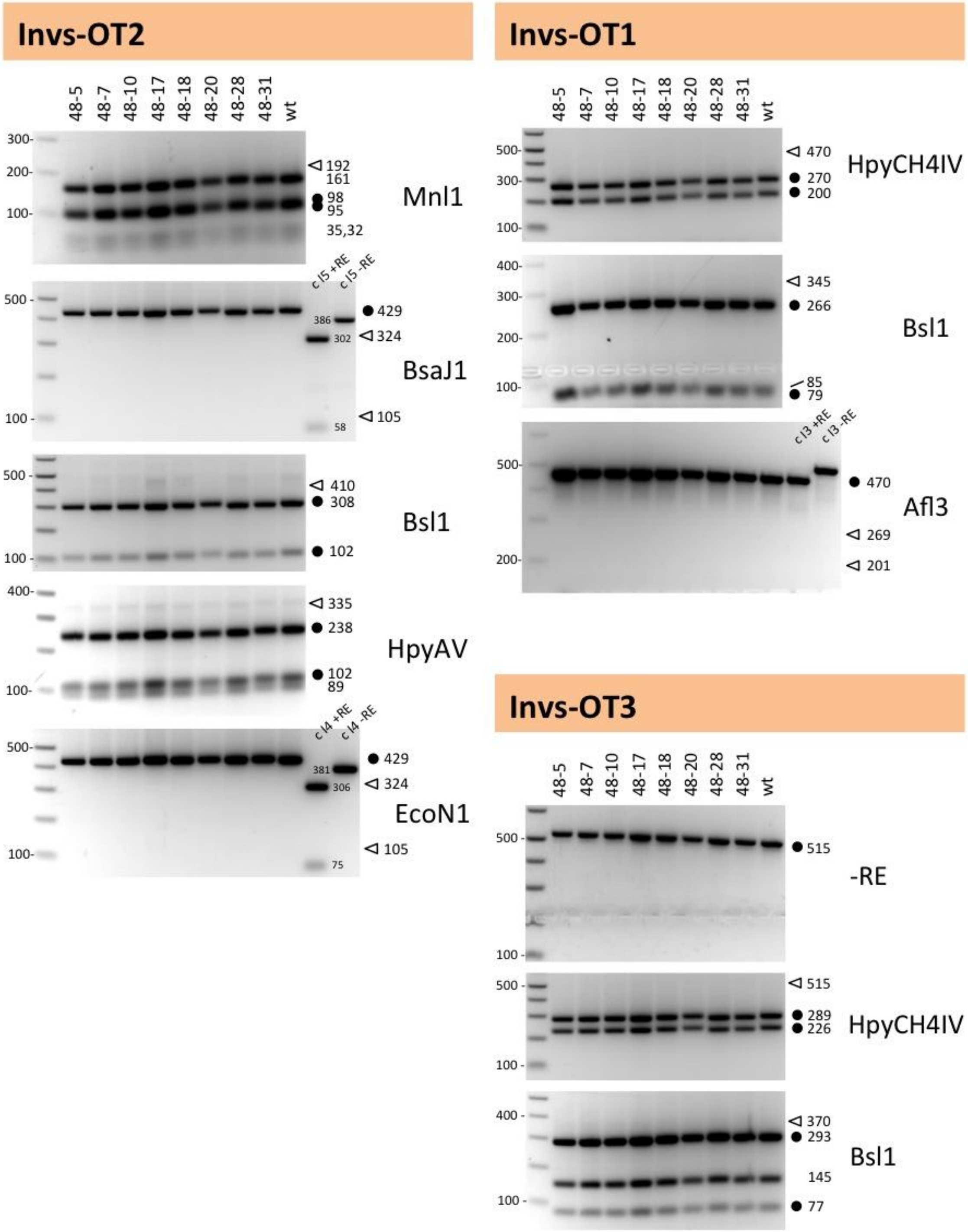

Appendix Figure S6 continued

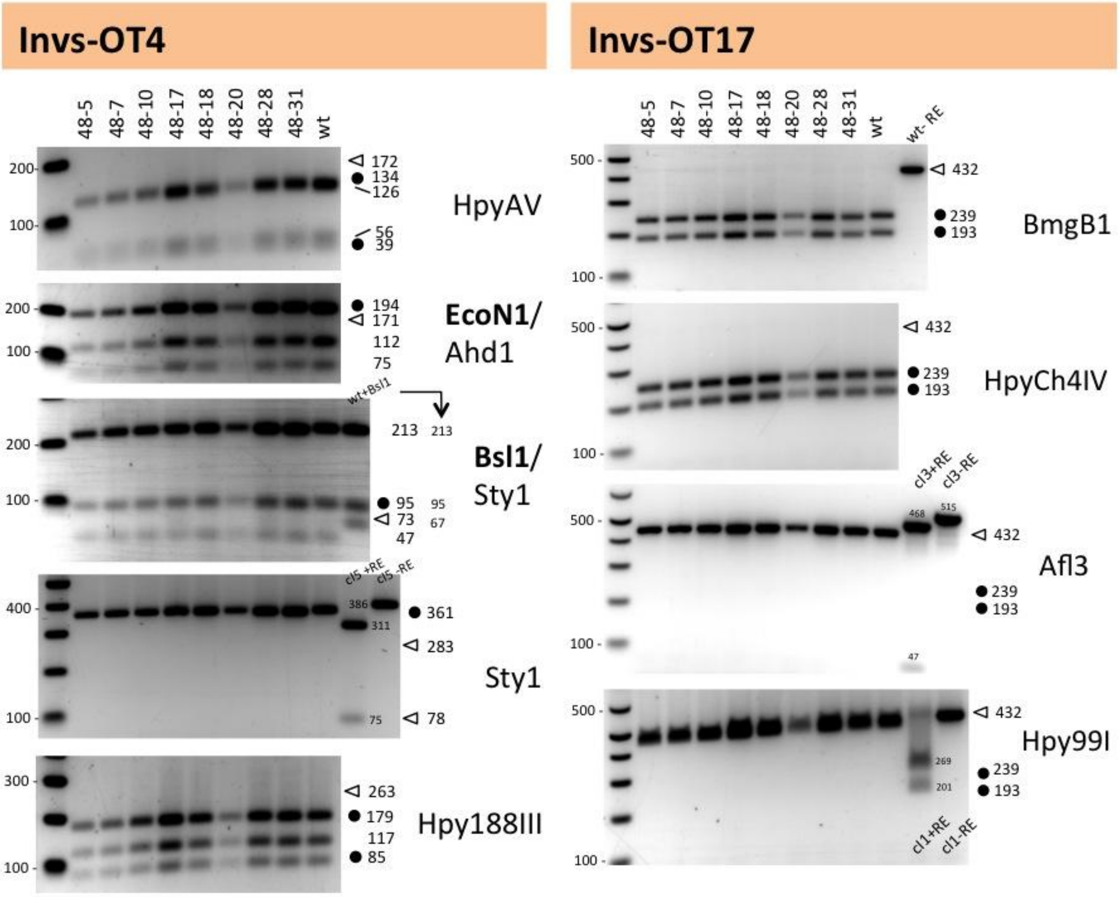

Appendix Figure S6 continued

Invs-OT5, original

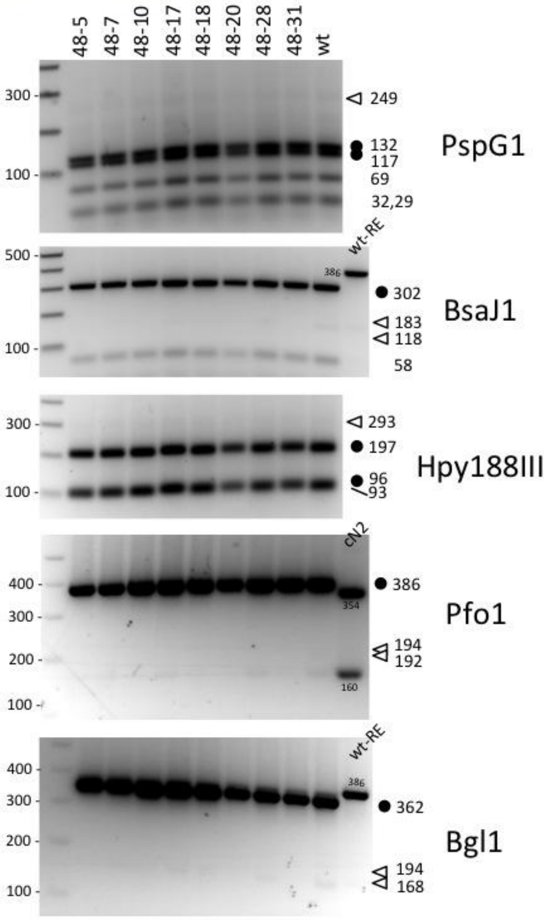

Invs-OT6, original

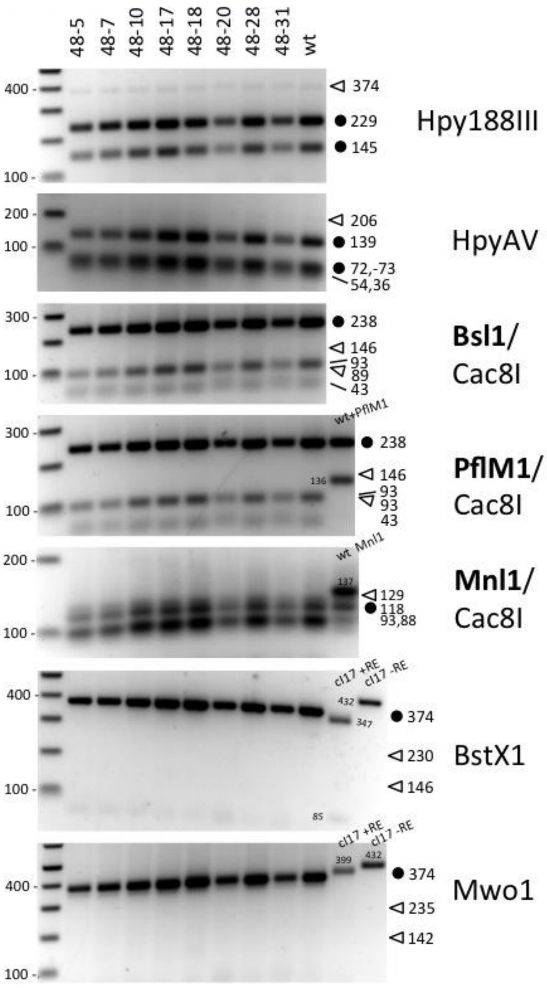

Invs-OT7

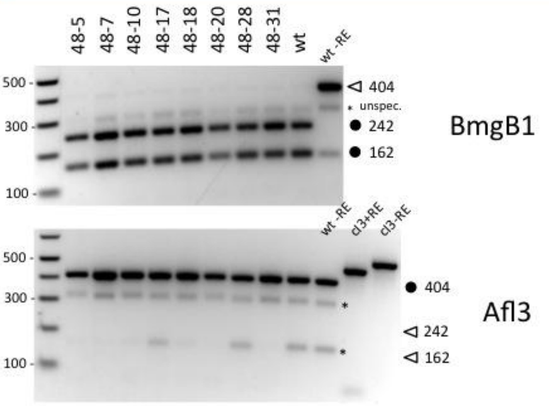

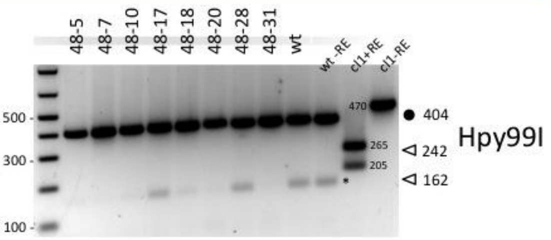

Appendix Figure S7

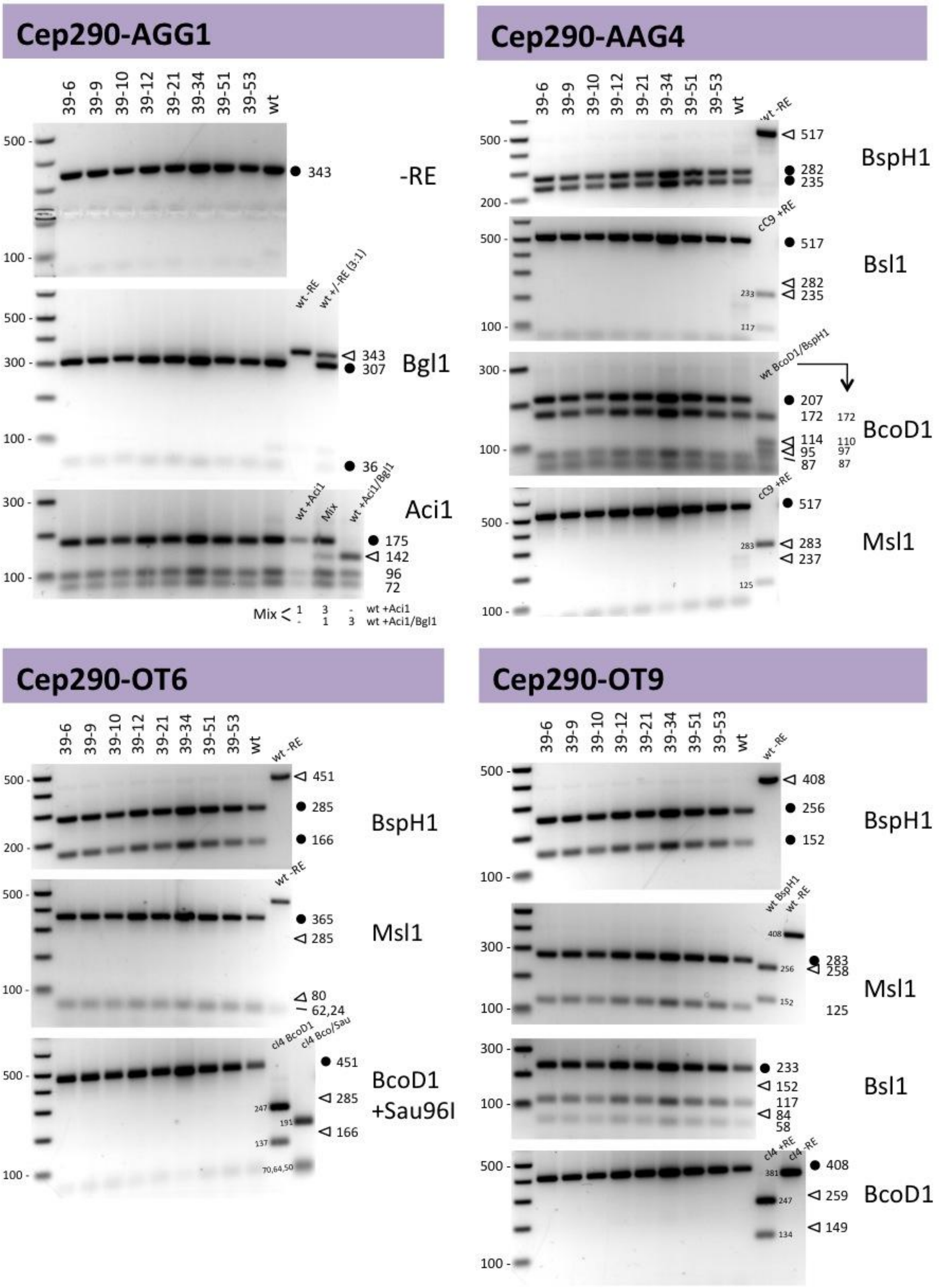

Appendix Figure S7 continued

Cep290-OT3

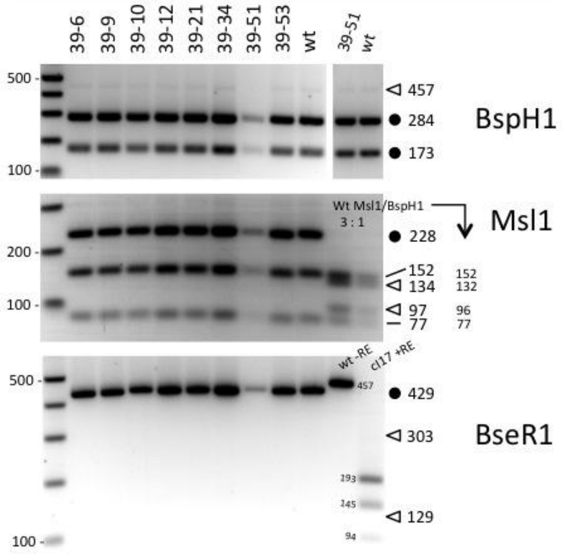

Cep290-OT4

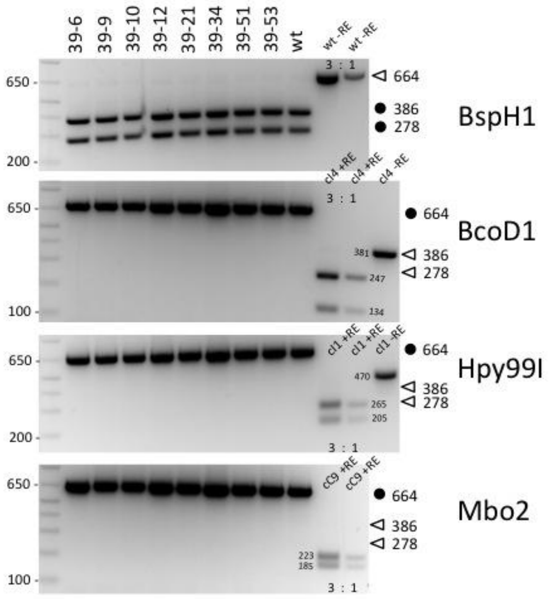

Cep290-OT5

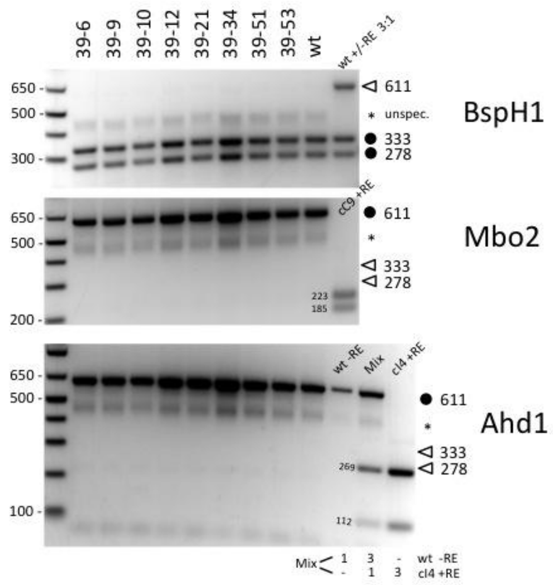

Appendix Figure S7 continued

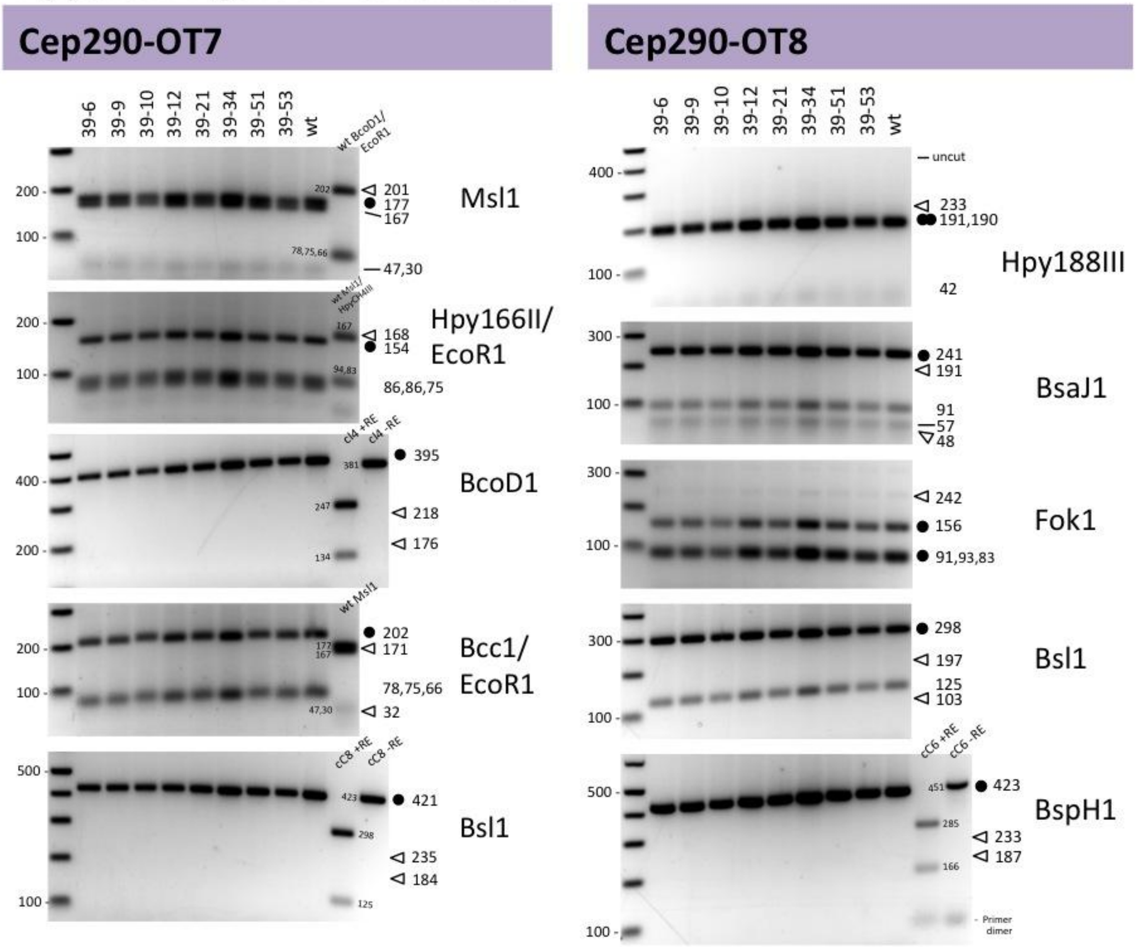

Appendix Figure S8

Nphp1-OT1

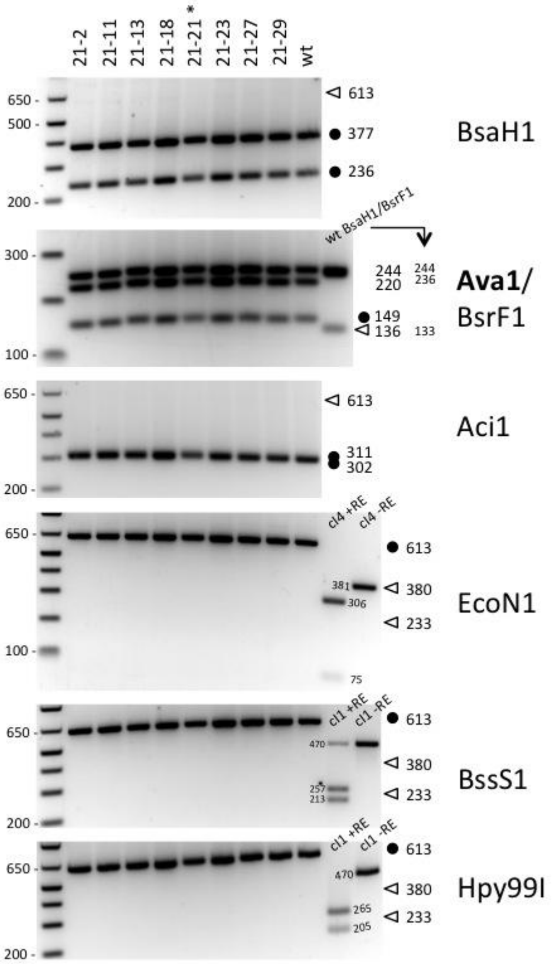

Nphp1-OT2

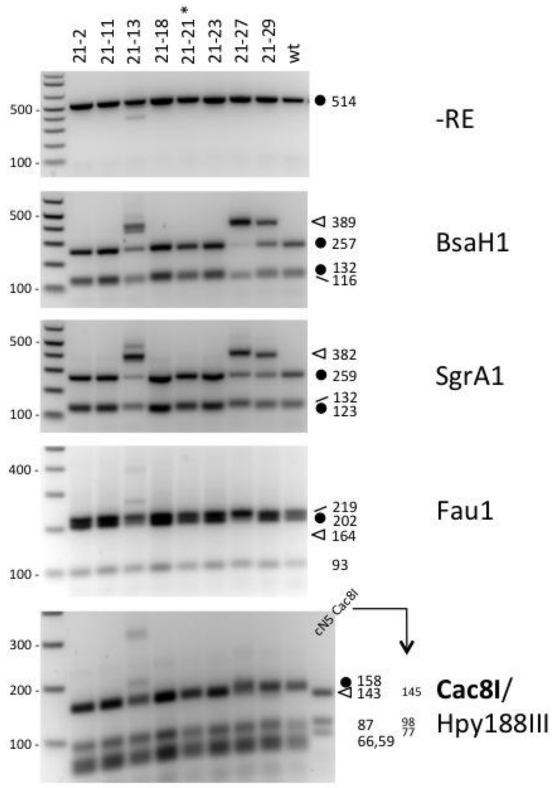

Nphp1-OT6

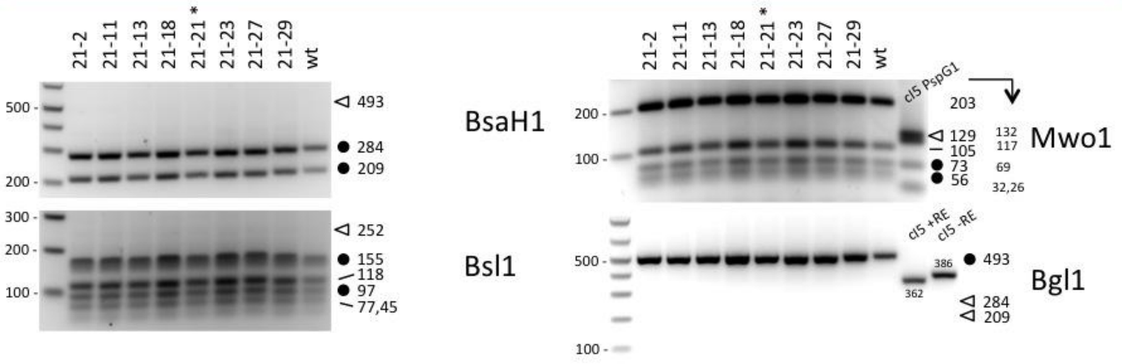

Appendix Figure S8 continued

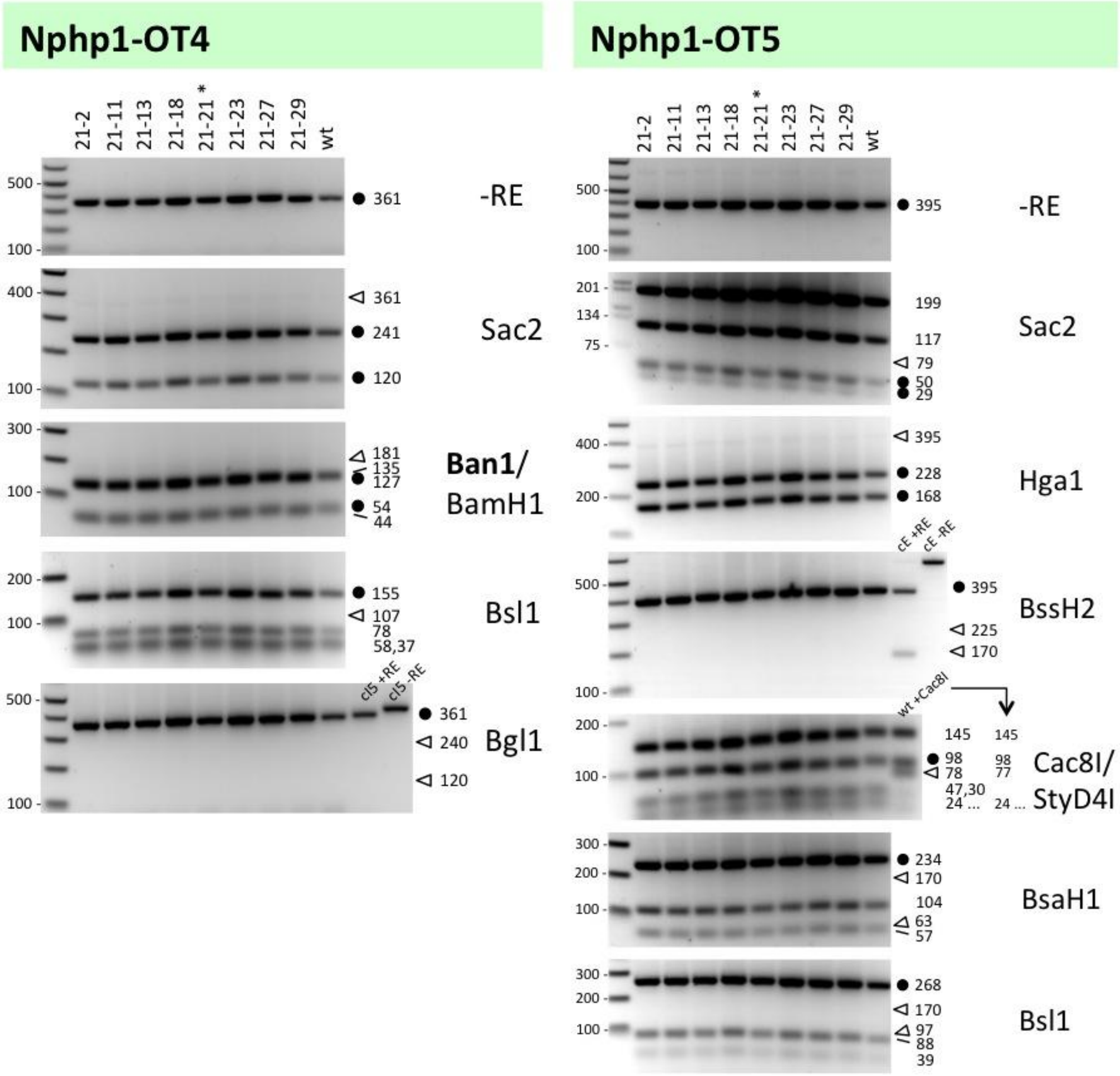

Appendix Figure S8 continued

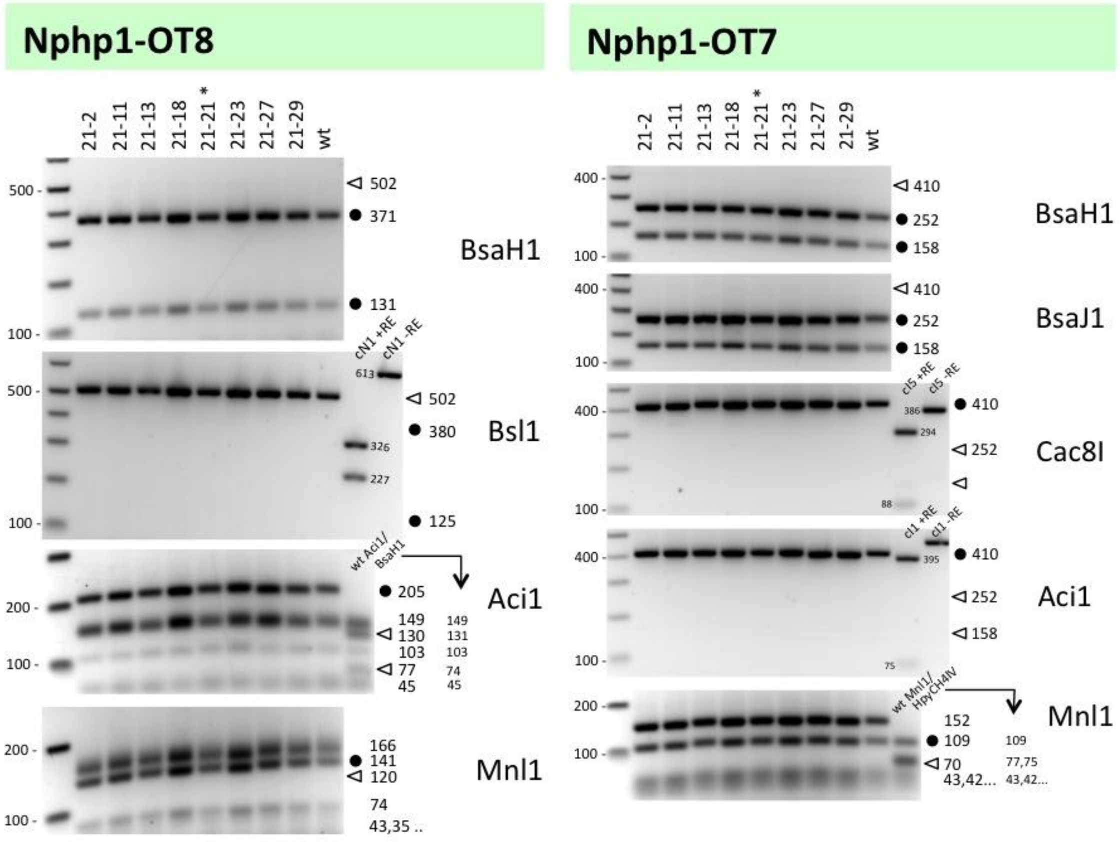

Appendix Figure S4-S8. **Screening clones for mutations in off-targets by RFLP analysis.** Off-target sites were amplified, and aliquots of the PCR products were restricted with the indicated enzymes and analysed by agarose gel electrophoresis. For each off-target site we analysed 6-8 clones (clone names are indicated at the top) and a WT sample. In a number of cases control samples were analysed in parallel. Control samples were either WT-PCR products amplified for the same off-target, or PCR products amplified for irrelevant off-targets (**C**xyz). Controls were included (WT +/- RE, or Cxyz +/-RE) either to demonstrate activity of the restriction enzyme (RE) in the reaction master mix, or to demonstrate that small size differences of closely sized DNA fragments can be resolved, or to demonstrate the sensitivity of detection. Relevant DNA fragments indicative for the WT configuration are marked with black circles, the position of DNA fragments indicative for the mutant configuration are indicated by open white triangles. In some cases double digests were performed to eliminate a DNA fragment co-migrating with an expected mutant fragment of similar size (the diagnostic enzyme is shown in boldface). The order of RFLP analyses shown for a given off-target displays at the top such enzyme restrictions capable in detecting the largest set of potential indel mutations.

Appendix Figure S9

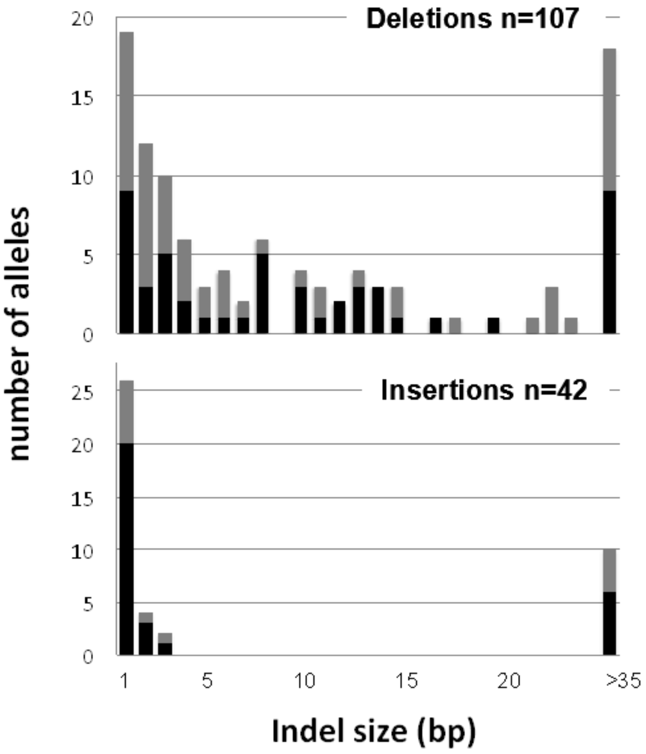

Appendix Figure S9. **Size distribution of indel mutations from sequenced on-targets.**

Collected data from 5 targeted loci (this study, n=81) are represented by black bars, and data from 4 additonal loci (data not shown, n=68), are represented by grey bars.

Four major categories of indel sizes are observed:

1. Deletions of 1-3 bps (27,5%, 41/149);
2. Deletions of 4-15 bps (26,8%, 40/149);
3. Insertions of 1 bp (17,4%, 26/149);
4. Indels >35 bps (18.8%, 28/149)

Appendix Figure S10

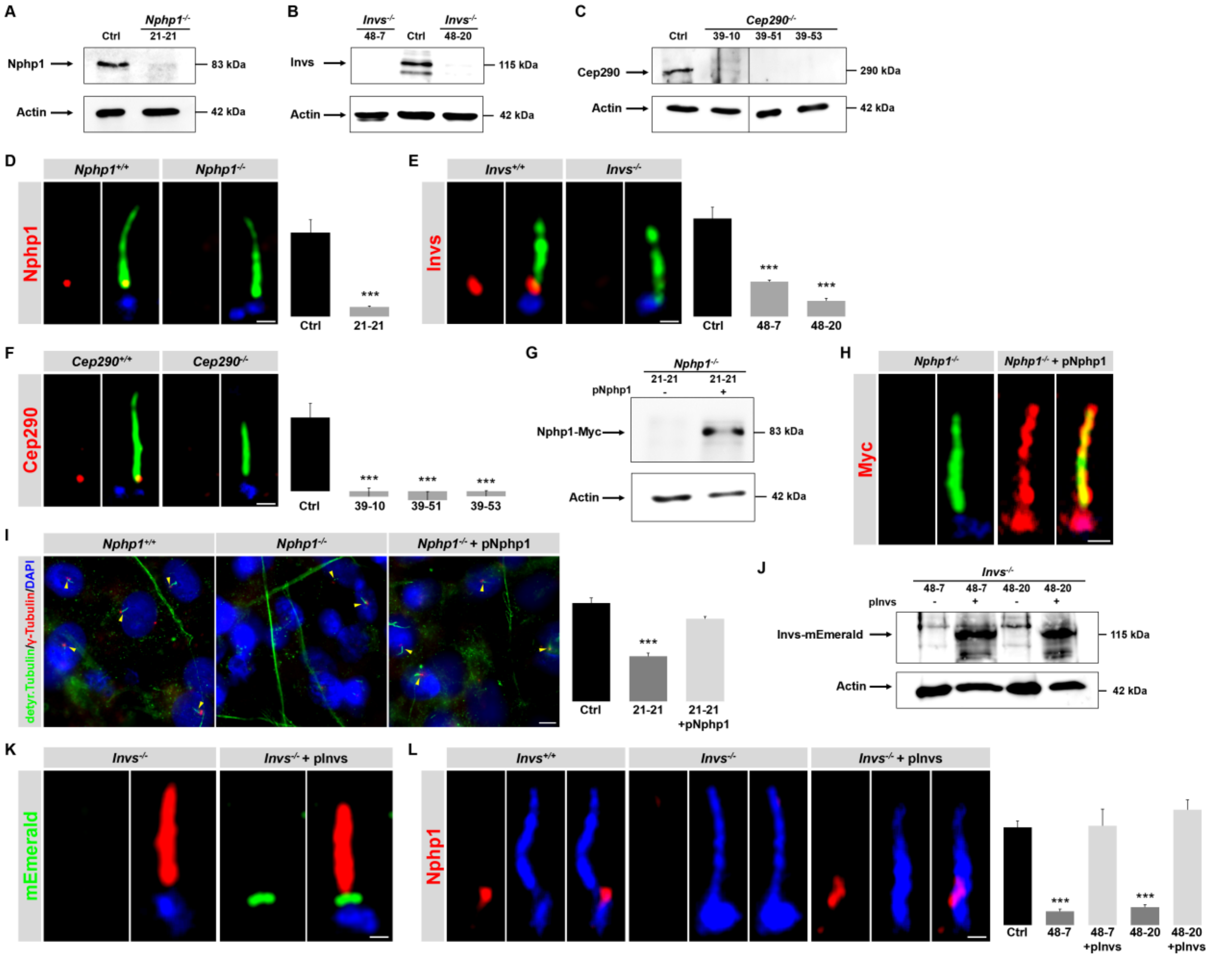

Appendix Figure S10. Confirmation of the inactivation of Nphp1 in *Nphp1*$^{-/-}$ NIH3T3 cells, of Invs in *Invs*$^{-/-}$ NIH3T3 cells and of Cep290 in *Cep290*$^{-/-}$ NIH3T3 cells. (A-C) Western blot studies with lysates obtained from *Nphp1*$^{-/-}$, *Invs*$^{-/-}$ and *Cep290*$^{-/-}$ NIH3T3 cells. Actin serves as loading control. (B, C, E, F, J-L) Different mutations of *Invs* and *Cep290* led to several clones of which each is genetically unique. (D-F) Immunofluorescence on WT NIH3T3 cells, on *Nphp1*$^{-/-}$ NIH3T3 cells, on *Invs*$^{-/-}$ NIH3T3 cells and on *Cep290*$^{-/-}$ NIH3T3 cells. The ciliary axoneme is stained in green by detyrosinated α-tubulin (D) or by acetylated α-tubulin (E and F) and the BB in blue by γ-tubulin. All measured proteins are shown in red. The black bars represent the normalised quantification in the WT and the grey bars the quantification in the mutants. Data are shown as mean ± s.e.m. The scale bars (in white)

represent a length of 0.5 μm. (G) Western blot analysis of lysates obtained from *Nphp1*$^{-/-}$ NIH3T3 cells and from *Nphp1*$^{-/-}$ NIH3T3 cells transfected with a plasmid encoding a Nphp1 (full-length)-Myc fusion protein. Actin serves as loading control. (H) Immunofluorescence on *Nphp1*$^{-/-}$ NIH3T3 cells and on *Nphp1*$^{-/-}$ NIH3T3 cells transfected with a plasmid encoding a Nphp1 (full-length)-Myc fusion protein. The ciliary axoneme is stained in green by acetylated α-tubulin and the BB in blue by γ-tubulin. Due to strong overexpression, the fusion protein (in red) localises to the whole cilium. The scale bar (in white) represents a length of 0.5 μm. (I) Immunofluorescence-based quantification of ciliated cells in WT NIH3T3 cells, in *Nphp1*$^{-/-}$ NIH3T3 cells and in *Nphp1*$^{-/-}$ NIH3T3 cells transfected with a plasmid encoding a Nphp1 (full-length)-Myc fusion protein. The following cell numbers were used for quantification: WT: 278 cells; *Nphp1*$^{-/-}$: 305 cells; *Nphp1*$^{-/-}$ expressing Nphp1-Myc: 275 cells. The black bar represents the normalised quantification in the WT, the dark grey bar the quantification in *Nphp1*$^{-/-}$ NIH3T3 cells and the bright grey bar the quantification in *Nphp1*$^{-/-}$ NIH3T3 cells transfected with a plasmid encoding a Nphp1 (full-length)-Myc fusion protein. Data are shown as mean ± s.e.m. The scale bar (in white) represents a length of 10 μm. (J) Western blot analysis of lysates obtained from *Invs*$^{-/-}$ NIH3T3 cells and from *Invs*$^{-/-}$ NIH3T3 cells transfected with a plasmid encoding a Invs (full-length)-mEmerald fusion protein. Actin serves as loading control. (K) Immunofluorescence on *Invs*$^{-/-}$ NIH3T3 cells and on *Invs*$^{-/-}$ NIH3T3 cells transfected with a plasmid encoding a Invs (full-length)-mEmerald fusion protein (in green). The ciliary axoneme is stained in red by acetylated α-tubulin and the BB in blue by γ-tubulin. The scale bar (in white) represents a length of 0.5 μm. (L) Immunofluorescence on WT NIH3T3 cells, on *Invs*$^{-/-}$ NIH3T3 cells and on *Invs*$^{-/-}$ NIH3T3 cells transfected with a plasmid encoding a Invs (full-length)-mEmerald fusion protein. The ciliary axoneme is stained in blue by detyrosinated α-tubulin and the BB in blue by γ-tubulin

(because the green channel is occupied by the green mEmerald signal). The scale bar (in white) represents a length of 0.5 μm. Nphp1 (in red) was quantified. At least, 20 cilia per individual were used for quantification. The black bar represents the normalised quantification in WT MEFs, the dark grey bar the quantification in *Invs*$^{-/-}$ NIH3T3 cells and the bright grey bar the quantification in *Invs*$^{-/-}$ NIH3T3 cells transfected with a plasmid encoding a Invs (full-length)-mEmerald fusion protein. Data are shown as mean ± s.e.m. (D) Asterisks denote statistical significance according to unpaired Student's *t*-tests (* $P<0.05$; ** $P<0.01$; *** $P<0.001$). (E, F, I, L) Asterisks denote statistical significance according to one-way ANOVA and Tukey HSD tests (* $P<0.05$; ** $P<0.01$; *** $P<0.001$) (E: $F(2,47)=35,20$, $P<0,0001$; F: $F(3,73)=16,34$, $P<0,0001$; I: $F(2,25)=51,17$, $P<0,0001$; L: $F(4,88)=40,94$, $P<0,0001$).

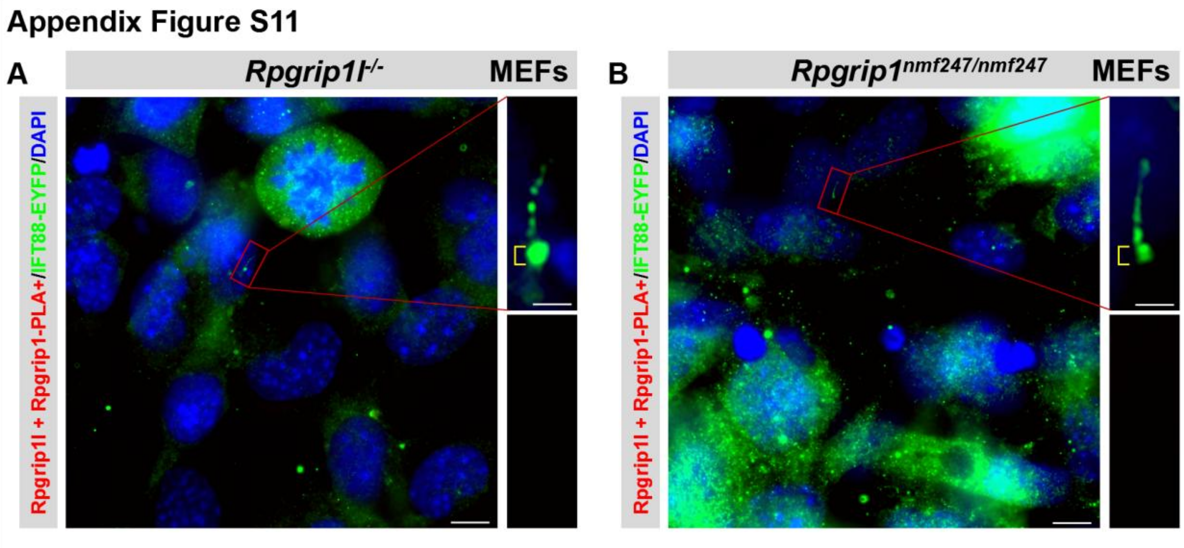

Appendix Figure S11. No PLA signal was detected in *Rpgrip1l*$^{-/-}$ and in *Rpgrip1*$^{nmf247/nmf247}$ MEFs. (A and B) In situ proximity ligation assay (in situ PLA) on *Rpgrip1l*$^{-/-}$ (A) and *Rpgrip1*$^{nmf247/nmf247}$ (B) MEFs. Cell nuclei are marked by DAPI (blue) and the ciliary axoneme by transiently transfected Ift88-EYFP (green). In the magnification, additional accumulation of Ift88-EYFP at the ciliary base is highlighted by yellow brackets. Scale bars (in white) depict 10 µm (overview) and 1 µm (magnification).

Appendix Figure S12

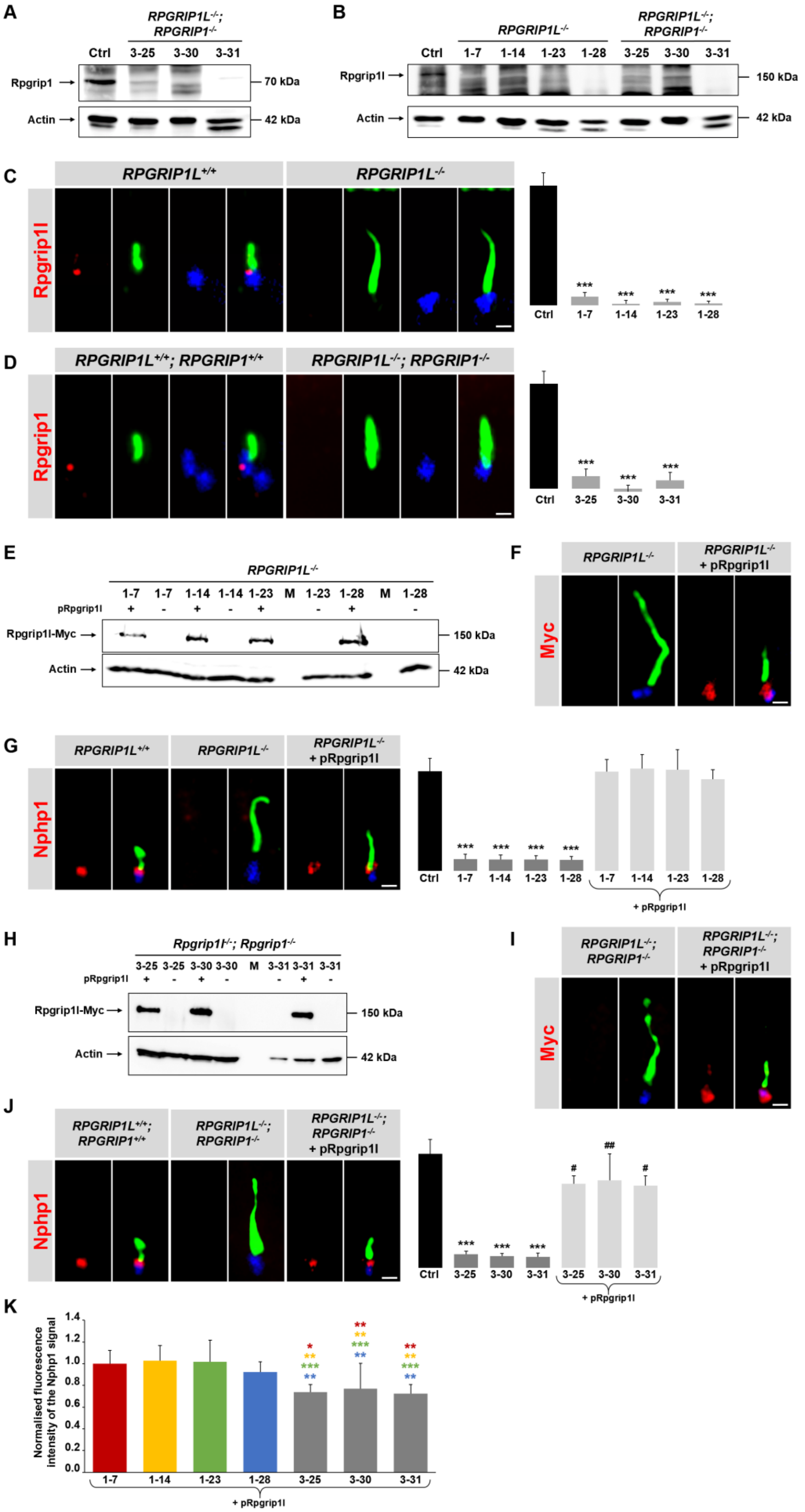

Appendix Figure S12. Characterisation of *RPGRIP1L*$^{-/-}$ and *RPGRIP1L*$^{-/-}$; *RPGRIP1*$^{-/-}$ HEK293 cell clones. (A and B) Western blot analysis of Rpgrip1 (A) and Rpgrip1l (B) in lysates from WT (Ctrl), *RPGRIP1L*$^{-/-}$ and *RPGRIP1L*$^{-/-}$; *RPGRIP1*$^{-/-}$ HEK293 cells. Different mutations of *RPGRIP1L* and *RPGRIP1* led to several clones of which each is genetically unique. Actin serves as loading control. (C and D) Immunofluorescence on WT, *RPGRIP1L*$^{-/-}$ (C) and *RPGRIP1L*$^{-/-}$; *RPGRIP1*$^{-/-}$ (D) HEK293 cells. Scale bars (in white) represent a length of 0.5 µm. The ciliary axoneme is stained in green by acetylated α-tubulin and the BB in blue by γ-tubulin. Rpgrip1l (C) and Rpgrip1 (D) are shown in red. At least, 10 cilia per individual were used for quantification. The black bars represent the normalised quantification in WT (Ctrl) HEK293 cells and the grey bars the quantifications in (C) *RPGRIP1L*$^{-/-}$ HEK293 cell clones and (D) *RPGRIP1L*$^{-/-}$; *RPGRIP1*$^{-/-}$ HEK293 cell clones. Statistical data are shown as mean ± s.e.m. Asterisks denote statistical significance according to one-way ANOVA and Tukey HSD tests (*** $P<0.001$) (C: $F(4,95)=59{,}94$, $P<0.0001$; D: $F(3,76)=30{,}97$, $P<0.0001$). (E and H) Western blot analysis of lysates from (E) *RPGRIP1L*$^{-/-}$ and (H) *RPGRIP1L*$^{-/-}$; *RPGRIP1*$^{-/-}$ HEK293 cell clones. Cells were transfected with a plasmid containing human full-length Rpgrip1l with a Myc-Tag (pRpgrip1l). Actin serves as loading control. (F and I) Immunofluorescence on (F) *RPGRIP1L*$^{-/-}$ and transfected *RPGRIP1L*$^{-/-}$ HEK293 cell clones and (I) *RPGRIP1L*$^{-/-}$; *RPGRIP1*$^{-/-}$ and transfected *RPGRIP1L*$^{-/-}$; *RPGRIP1*$^{-/-}$ HEK293 cell clones. Scale bars (in white) represent a length of 0.5 µm. The ciliary axoneme is stained in green by acetylated α-tubulin and the BB in blue by γ-tubulin. Myc is shown in red. (G and J) Immunofluorescence on (G) WT (Ctrl), *RPGRP1L*$^{-/-}$ and transfected *RPGRIP1L*$^{-/-}$ HEK293 cells and (J) WT (Ctrl), *RPGRIP1L*$^{-/-}$; *RPGRIP1*$^{-/-}$ and transfected *RPGRIP1L*$^{-/-}$; *RPGRIP1*$^{-/-}$ HEK293 cells. Scale bars (in white) represent a length of 0.5 µm. The ciliary axoneme is stained in green by detyrosinated α-tubulin and the BB in blue by γ-tubulin. Nphp1 is shown in red. The black bars represent the normalised quantification in WT (Ctrl) HEK293 cells, the

dark grey bars the quantification in (G) *RPGRIP1L*$^{-/-}$ or (J) *RPGRIP1L*$^{-/-}$; *RPGRIP1*$^{-/-}$ HEK293 cell clones. The bright grey bars represent the quantifications in (G) transfected *RPGRIP1L*$^{-/-}$ or (J) transfected *RPGRIP1L*$^{-/-}$; *RPGRIP1*$^{-/-}$ HEK293 cell clones. Statistical Data are shown as mean ± s.e.m. Asterisks indicate significance in comparison to the WT, while hash signs represent significance in comparison to the WT and the *RPGRIP1L*$^{-/-}$; *RPGRI1P1*$^{-/-}$ cell clones according to one-way ANOVA and Tukey HSD tests (*** P<0.001, # P<0,05, ## P<0,01) (F(14,116)=32,42 P<0.0001). (K) Normalised fluorescence intensity of the Nphp1 signal in *RPGRIP1L*$^{-/-}$ and *RPGRIP1L*$^{-/-}$; *RPGRIP1*$^{-/-}$ HEK293 cell clones. The coloured bars represent the quantifications in *RPGRIP1L*$^{-/-}$ HEK293 cell clones and the grey bars the quantifications in *RPGRIP1L*$^{-/-}$; *RPGRIP1*$^{-/-}$ HEK293 cell clones. Statistical data are shown as mean ± s.e.m. Asterisks denote statistical significance according to one-way ANOVA and Tukey HSD tests (* P<0,05, ** P<0,01, *** P<0.001) (F(14,116)=32,42 P<0.0001). Asterisks colour indicates significant differences between several *RPGRIP1L*$^{-/-}$ HEK293 cell clones and *RPGRIP1L*$^{-/-}$; *RPGRIP1*$^{-/-}$ HEK293 cell clones.

Appendix Figure S13

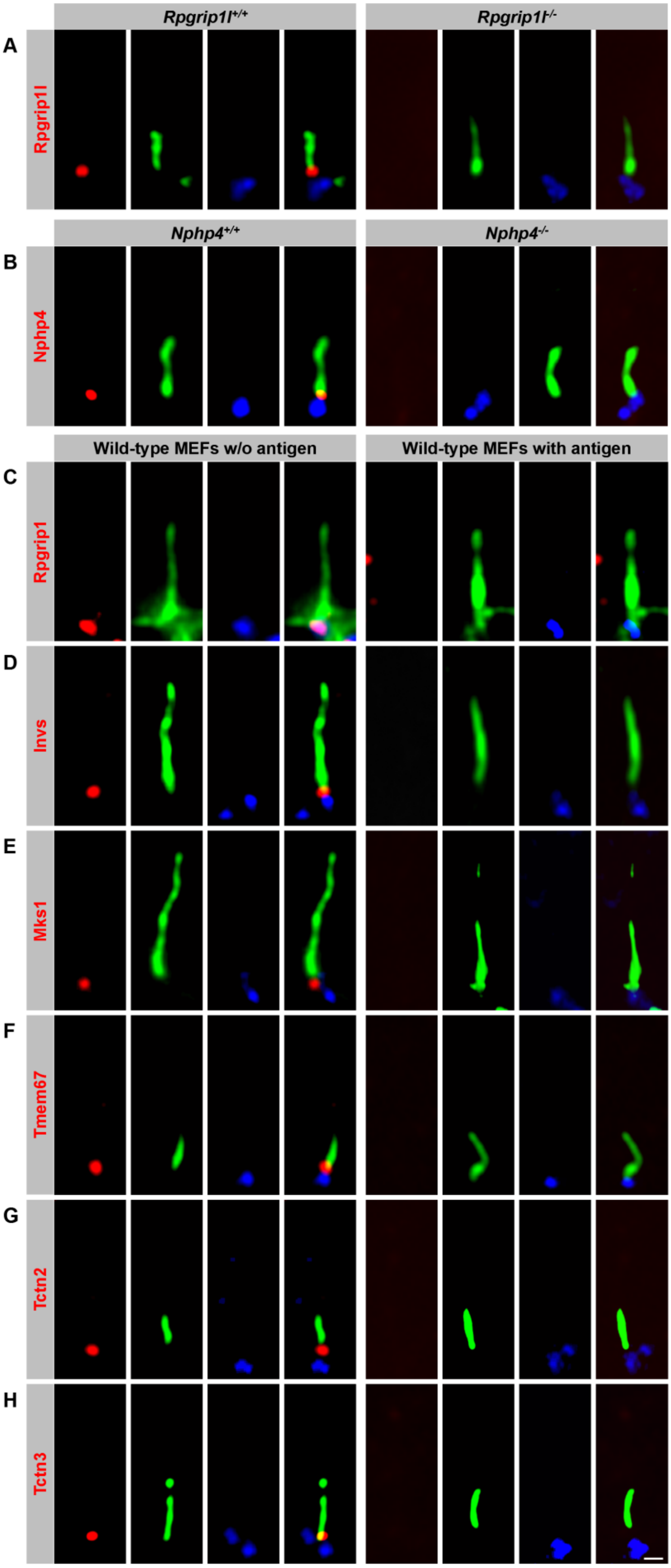

Appendix Figure S13. Illustration of antibody specificity. Immunofluorescence on MEFs obtained from WT (A-H), *Rpgrip1l*$^{-/-}$ (A) and *Nphp4*$^{-/-}$ (B) embryos. The axoneme is marked by acetylated α-tubulin (green) and the basal body by γ-tubulin (blue). All proteins of interest are marked by their specific antibody (red). (C-H) The stainings were performed in combination with the appropriate antigen. The scale bar (in white) depicted in (H) represents a length of 0.5 µm and also applies for (A-G).

Appendix Table S1.		Screening clones for mutations in off-targets by RFLP analysis							
			number of possible deletions not detectable				number of possible insertions not detectable		
Off-target	MMs[a]	Clones with mutations detected	Del-1 n=2[b]	Del-2 n=3[b]	Del-3 n=4[b]	Del 4-15 n=126[b]	Ins+1 n=4[b]	Ins+2 n=16[b]	Ins+3 n=64[b]
RPGRIP1L-OT-cx1	3	0/6	none	none	none	none	none	none	none
RPGRIP1L-OT1	3	0/6	none	none	none	7	3	none	35
RPGRIP1L-OT-cx2	4	0/6	none	none	none	none	1	1	4
RPGRIP1L-OT2	4	0/6***	1	2	none	none	3	none	39
RPGRIP1L-OT3	4	0/6	none	none	none	3	none	none	41
RPGRIP1L-OT4	3	0/6	none	none	none	1	none	none	41
RPGRIP1L-OT-cx3	4	0/6	none	none	none	none	none	none	none
RPGRIP1L-OT5	3	0/6	none	none	none	none	none	1	4
RPGRIP1L-OT-cx4	3	0/6	none	none	none	4	none	none	37
RPGRIP1L-OT-cx6	3	0/6	none	none	none	6	2	10	none
RPGRIP1L-OT-cx7	4	0/6	none	none	none	none	none	none	none
RPGRIP1-OT1	4	0/8	none	none	none	none	none	1	5
RPGRIP1-OT2	4	0/8	none	none	none	1	none	1	5
RPGRIP1-OT3	4	0/8	none	none	none	none	none	1	5
RPGRIP1-OT4	4	0/8	none	none	1	none	none	1	5
RPGRIP1-OT5	4	0/8	none	none	none	none	none	1	5
Invs-OT2	2	0/8	none	1	none	none	1	2	7
Invs-OT1	2	0/8	none	none	none	1	none	none	1
Invs-OT3	3	0/8	none	none	none	none	none	none	1
Invs-OT4	3	0/8***	2	3	none	30	none	not cal.[c]	not cal.
Invs-OT17	4	0/8	none	none	none	none	none	none	1
Invs-OT5	3	0/8	none	none	none	none	none	1	4
Invs-OT6	3	0/8	none	none	none	none	none	none	13
Invs-OT7	3	0/8	none	none	none	none	none	none	1
Cep290-AGG1	2	0/8	none	none	none	none	none	none	none
Cep290-AAG4	3	0/8	none	none	none	1	none	none	2
Cep290-OT6	3	0/8	none	none	none	none	none	none	2
Cep290-OT9	4	0/8	none	none	none	1	none	none	2
Cep290-OT3	3	0/8	none	none	none	none	none	1	2
Cep290-OT4	4	0/8	none	none	2	2	none	none	3
Cep290-OT5	3	0/8	none	1	none	1	none	1	3
Cep290-OT7	3	0/8***	1	none	4	>20	3	14	42
Cep290-OT8	3	0/8	none	none	none	2	none	none	none
Nphp1-OT1	2	0/7	none	none	none	1	none	none	5
Nphp1-OT2	3	3/7***	none	none	4	6	none	3	16
Nphp1-OT6	3	0/7	none	none	none	1	1	none	none
Nphp1-OT4	3	0/7	none	none	none	1	none	2	none
Nphp1-OT5	3	0/7	none	none	none	none	none	3	12
Nphp1-OT8	3	0/7***	1	1	none	2	1	2	none
Nphp1-OT7	3	0/7***	1	none	none	none	1	2	8

[a] number of mismatches present in the off-target, [b] n=total number of different mutations possible, *** clones from which off-target alleles have been sequenced (Appendix Table S2), [c] not calculated

Appendix Table S2.

Sequence analysis of cloned off-target alleles		
**Off-target**	**clone**	**mutations detected/ samples sequenced**
RPGRIP1L-OT2	1-7	0/6
	1-14	0/6
	1-23	0/6
	1-28	0/6
Invs-OT4	48-7	0/6
	48-20	0/6
Cep290-OT7	39-51	0/6
	39-53	0/6
Nphp1-OT2	21-21	0/7
	21-23	0/8
Nphp1-OT8	21-21	0/8
	21-23	0/8
Nphp1-OT7	21-21	0/8

Appendix References

Arts H, Doherty D, van Beersum S, Parisi M, Letteboer S, Gorden N, Peters T, Märker T, Voesenek K, Kartono A, Ozyurek H, Farin F, Kroes H, Wolfrum U, Brunner H, Cremers F, Glass I, Knoers N, Roepman R (2007) Mutations in the gene encoding the basal body protein RPGRIP1L, a nephrocystin-4 interactor, cause Joubert syndrome. *Nat Genet* 39: 882-888.

Bae S, Park J, Kim J (2014) Cas-OFFinder: a fast and versatile algorithm that searches for potential off-target sites of Cas9 RNA-guided endonucleases. *Bioinformatics* 30: 1473-1475.

Hsu P, Scott D, Weinstein J, Ran F, Konermann S, Agarwala V, Li Y, Fine E, Wu X, Shalem O, Cradick T, Marraffini L, Bao G, Zhang F (2013) DNA targeting specificity of RNA-guided Cas9 nucleases. *Nat Biotechnol* 31: 827-832.

Leibiger C, Kosyakova N, Mkrtchyan H, Glei M, Trifonov V, Liehr T (2013) First molecular cytogenetic high resolution characterization of the NIH 3T3 cell line by murine multicolor banding. *J Histochem Cytochem* 61: 306-312.

Ran F, Hsu P, Wright J, Agarwala V, Scott D, Zhang F (2013) Genome engineering using the CRISPR-Cas9 system. *Nat Protoc* 8: 2281-2308.

Schou K, Mogensen J, Morthorst S, Nielsen B, Aleliunaite A, Serra-Marques A, Fürstenberg N, Saunier S, Bizet A, Veland I, Akhmanova A, Christensen S, Pedersen L (2017) KIF13B establishes a CAV1-enriched microdomain at the ciliary transition zone to promote Sonic hedgehog signalling. *Nat Commun* 8: 14177.

Yang H, Wang H, Shivalila C, Cheng A, Shi L, Jaenisch R (2013) One-step generation of mice carrying reporter and conditional alleles by CRISPR/Cas-mediated genome engineering. *Cell* 154: 1370-1379.
