# Supplementary material for: Cell type‐specific regulation of ciliary transition zone assembly in vertebrates
Source: EMBO J. 2018 Apr 12;37(10):e97791. doi: 10.15252/embj.201797791 (PMC5978567; doi:10.15252/embj.201797791)
Supplement: Supplementary file 2 — Expanded View Figures PDF [file EMBJ-37-e97791-s002.pdf]

Expanded View Figures

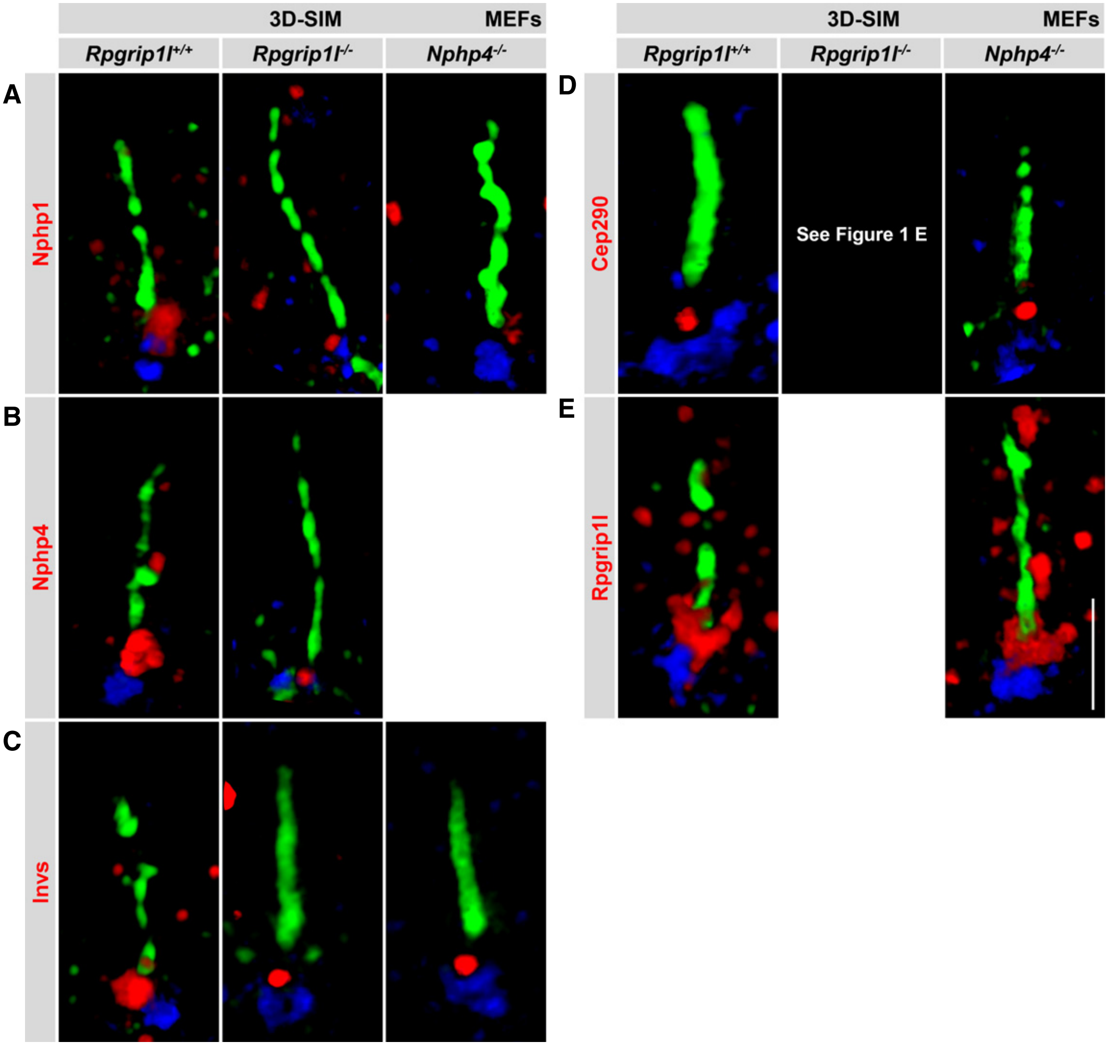

Figure EV1. Loss of Rpgrip1l or Nphp4 does not alter the localisation of the components of the Nphp modules.

A–E Immunofluorescence on MEFs which were obtained from WT, *Rpgrip1l*$^{-/-}$ and *Nphp4*$^{-/-}$ mouse embryos. Images were obtained by using super-resolution microscopy (3D-SIM). The ciliary axoneme is stained in green by detyrosinated α-tubulin (A) or by acetylated α-tubulin (B–E) and the BB in blue by γ-tubulin (A–E). (A–E) All TZ proteins are shown in red. The scale bar (in white) depicted in (E) represents a length of 2 μm and also applies for (A–D).

© 2018 The Authors

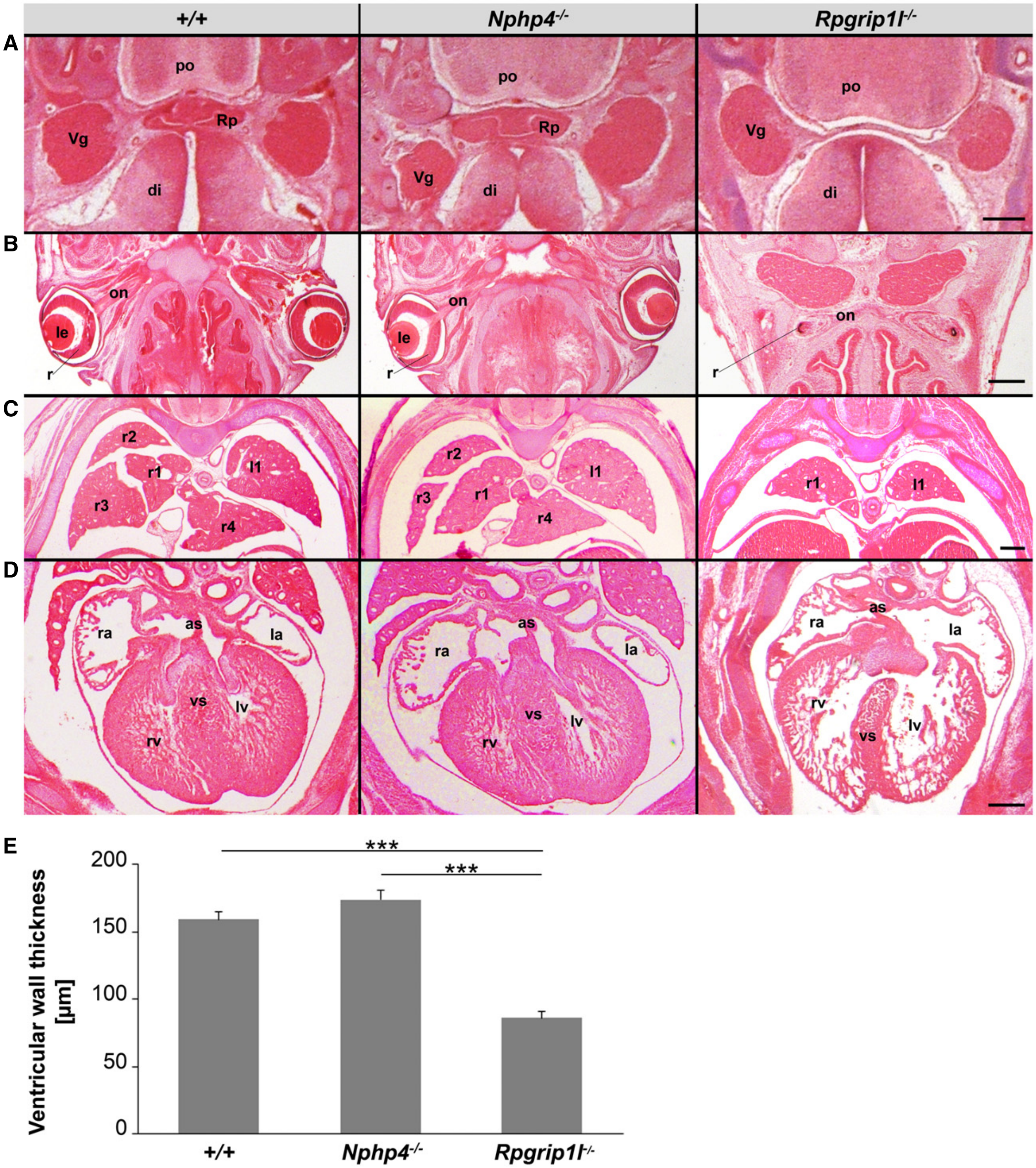

Figure EV2. In contrast to *Rpgrip1l*$^{-/-}$ mouse embryos, *Nphp4*$^{-/-}$ mouse embryos display a normal development of the Rathke's pouch, the eyes, the lung and the heart.

A–D Haematoxylin and eosin (H & E) staining of (A) the Rathke's pouch, (B) the eyes, (C) the lung and (D) the heart in WT, *Nphp4*$^{-/-}$ and *Rpgrip1l*$^{-/-}$ mouse embryos at E14.5. Scale bars (in black) represent a length of 250 µm. as, atrial septum; di, diencephalon; la, left atrium; le, lens; lv, left ventricle; l1, left lung lobe 1; on, optic nerve; po, pons; r, retina; ra, right atrium; rv, right ventricle; r1-4, right lung lobes 1-4; Rp, Rathke's pouch; Vg, trigeminal ganglion; vs, ventricular septum.

E Quantification of ventricular wall thickness in embryonic hearts of WT, *Nphp4*$^{-/-}$ and *Rpgrip1l*$^{-/-}$ mouse embryos. n = 3 of all three genotypes were used for these measurements. Data are shown as mean ± s.e.m. Asterisks denote statistical significance according to one-way ANOVA and Tukey HSD tests (***$P < 0.001$; $F(2, 30) = 64.05$, $P < 0.0001$).

© 2018 The Authors

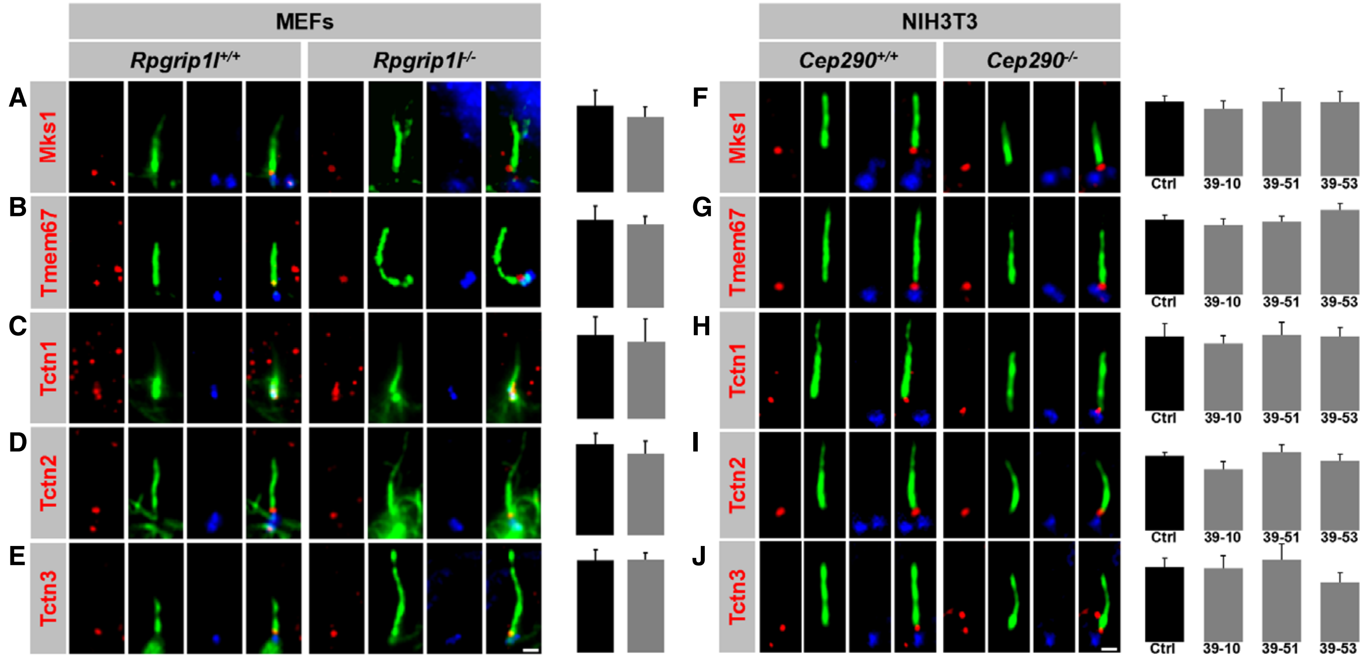

Figure EV3. The TZ amount of all analysed Mks/B9 module components is unaltered in *Rpgrip1l*$^{-/-}$ MEFs and *Cep290*$^{-/-}$ NIH3T3 cell clones.

A–J (A–E) Immunofluorescence on MEFs which were obtained from WT and *Rpgrip1l*$^{-/-}$ embryos. The scale bar (in white) depicted in (E) represents a length of 0.5 μm and also applies for (A–D). (F–J) Immunofluorescence on WT and *Cep290*$^{-/-}$ NIH3T3 cells. Different mutations of *Cep290* led to several clones of which each is genetically unique. The scale bar (in white) depicted in (J) represents a length of 0.5 μm and also applies for (F–I). (A–J) The ciliary axoneme is stained in green by acetylated α-tubulin and the BB in blue by γ-tubulin. All measured proteins are shown in red. (A–E) n = 5 embryos of both genotypes were used for these measurements, respectively. (A–J) At least 20 cilia per individual were used for quantification. The black bars represent the normalised quantification in WT MEFs (A–E) and WT (Ctrl) NIH3T3 cells (F–J) and the grey bars the quantifications in *Rpgrip1l*$^{-/-}$ MEFs (A–E) and *Cep290*$^{-/-}$ NIH3T3 cell clones (F–J), respectively. Data are shown as mean ± s.e.m. Statistical evaluation was performed by using unpaired Student's *t*-tests (A–E) and one-way ANOVA and Tukey HSD tests (F–J).

Figure EV4. Loss of Rpgrip1l results in ultrastructural alterations of the TZ and reduces the ciliary amount of Arl13b *in vitro* and *in vivo*.

A, B (A) Transmission electron microscopy of the TZ of WT and *Rpgrip1l*$^{-/-}$ mouse embryonic kidney cilia at E18.5. (B) Transmission electron microscopy of the TZ of WT and *Rpgrip1l*$^{-/-}$ mouse limb bud cilia at E12.5. (A and B) Almost all Y-linkers of the TZ of *Rpgrip1l*$^{-/-}$ embryonic kidney and limb bud cilia are barely or not detectable compared with the well visible ones in wild-type littermates. Some individual Y-linkers can be seen (indicated by red arrowheads) but seem to be substantial reduced. Furthermore, some of the axoneme microtubules do not exist as doublets but as singlets in the absence of Rpgrip1l (indicated by yellow arrowheads). Scale bars (in black) represent a length of 100 nm.

C–E Immunofluorescence on MEFs, kidneys (E18.5) and limb buds (E12.5) which were obtained from WT and *Rpgrip1l*$^{-/-}$ embryos. Scale bars (in white) represent a length of 0.5 μm. The ciliary axoneme is stained in green by acetylated α-tubulin and the BB in blue by γ-tubulin. Arl13b is shown in red. (C, E) n = 4 and (D) n = 3 of both genotypes were used for these measurements, respectively. At least 20 cilia per individual were used for quantification. The black bars represent the normalised quantification in WT MEFs, mouse embryonic kidneys and mouse embryonic limb buds and the grey bars the quantification in the *Rpgrip1l*$^{-/-}$ status. Data are shown as mean ± s.e.m. Statistical evaluation was performed by using unpaired Student's *t*-tests. Asterisks denote statistical significance (**P < 0.01; ***P < 0.001).

▶

© 2018 The Authors

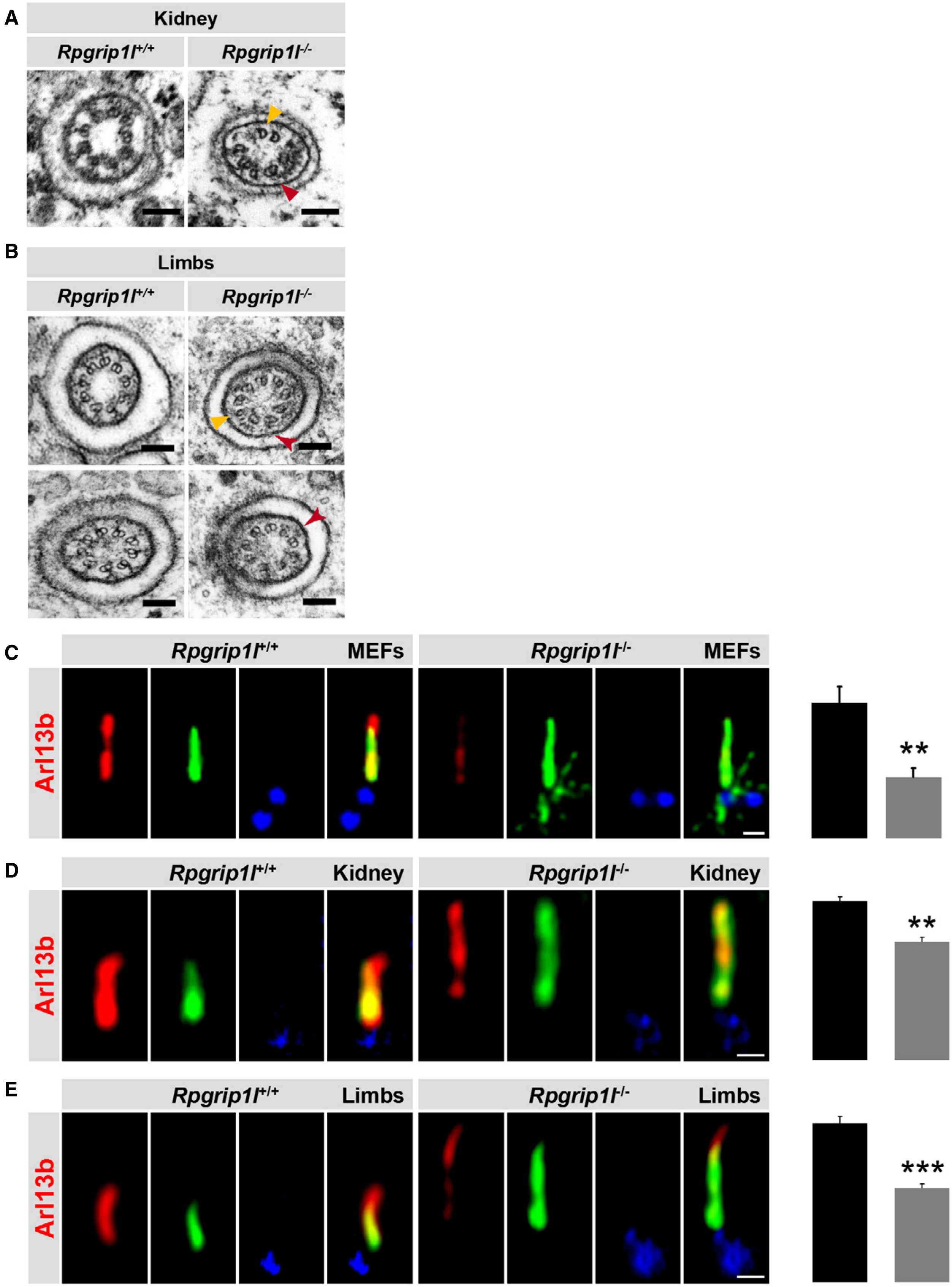

Figure EV4.

 © 2018 The Authors
