# Supplementary material for: Cell type‐specific regulation of ciliary transition zone assembly in vertebrates
Source: EMBO J. 2018 Apr 12;37(10):e97791. doi: 10.15252/embj.201797791 (PMC5978567; doi:10.15252/embj.201797791)
Supplement: Supplementary file 4 — Movie EV2 [file EMBJ-37-e97791-s004.zip › Movie_EV2_legend.rtf]

Movie EV2. Illustration of Cep290 at the TZ of a Rpgrip1l-negative cilium. Z-stacks were made via combining images which were taken by using super-resolution microscopy (3D-SIM). These z-stacks were reconstructed in 3D and presented in a Movie sequence. The ciliary axoneme is marked by acetylated -tubulin in green. The BB is marked by -tubulin in blue. The TZ protein Cep290 is depicted in red.
