# Supplementary figures and images for: Cell type‐specific regulation of ciliary transition zone assembly in vertebrates
Source: EMBO J. 2018 Apr 12;37(10):e97791. doi: 10.15252/embj.201797791 (PMC5978567; doi:10.15252/embj.201797791)

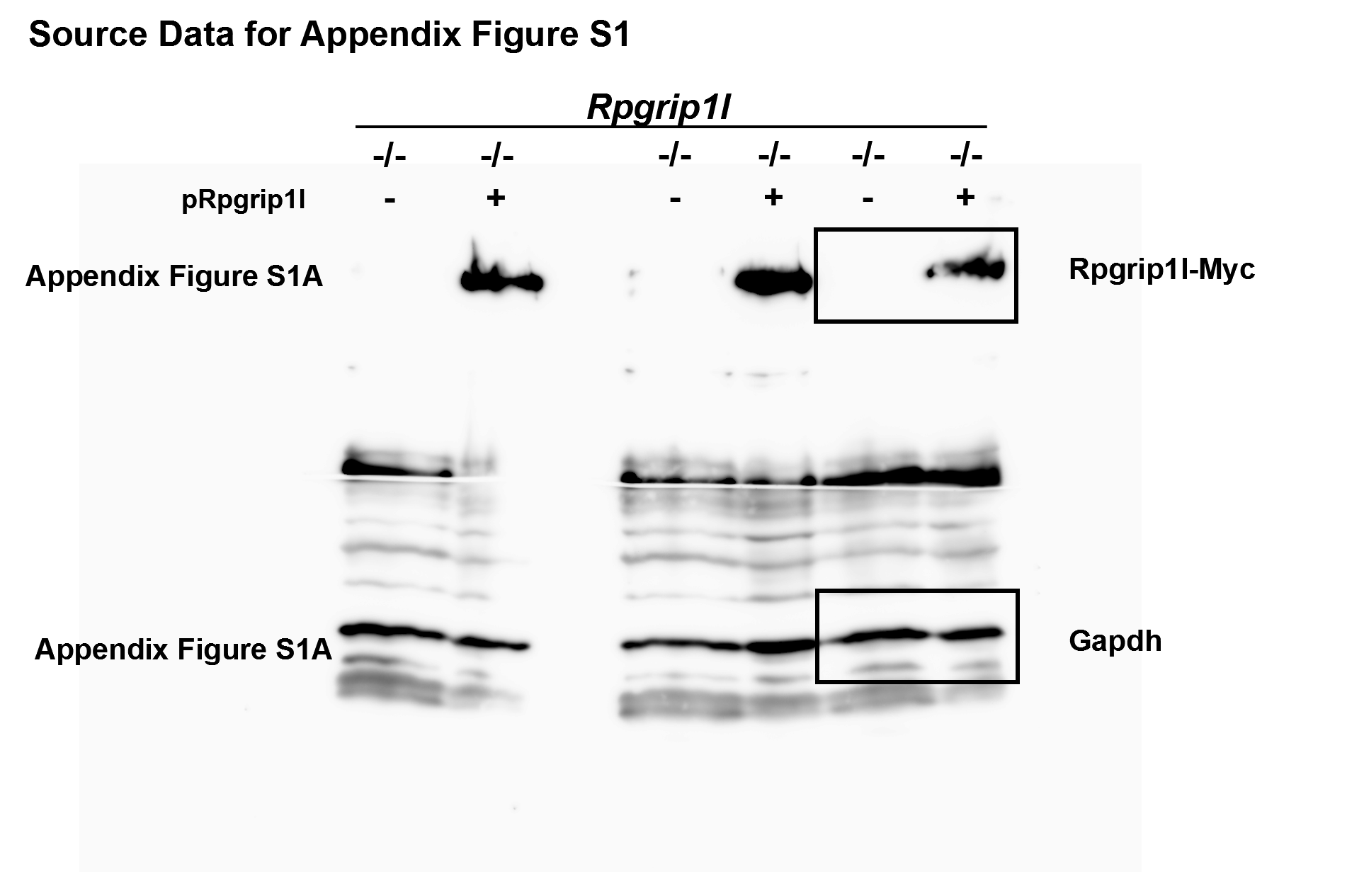

Supplement: Supplementary file 5 — Source Data for Appendix [file EMBJ-37-e97791-s008.zip › EMBOJ-2017-97791R_Appendix_Source_Data/EMBOJ-2017-97791R_Source_Data_for_Appendix_Figure_S1.tif]

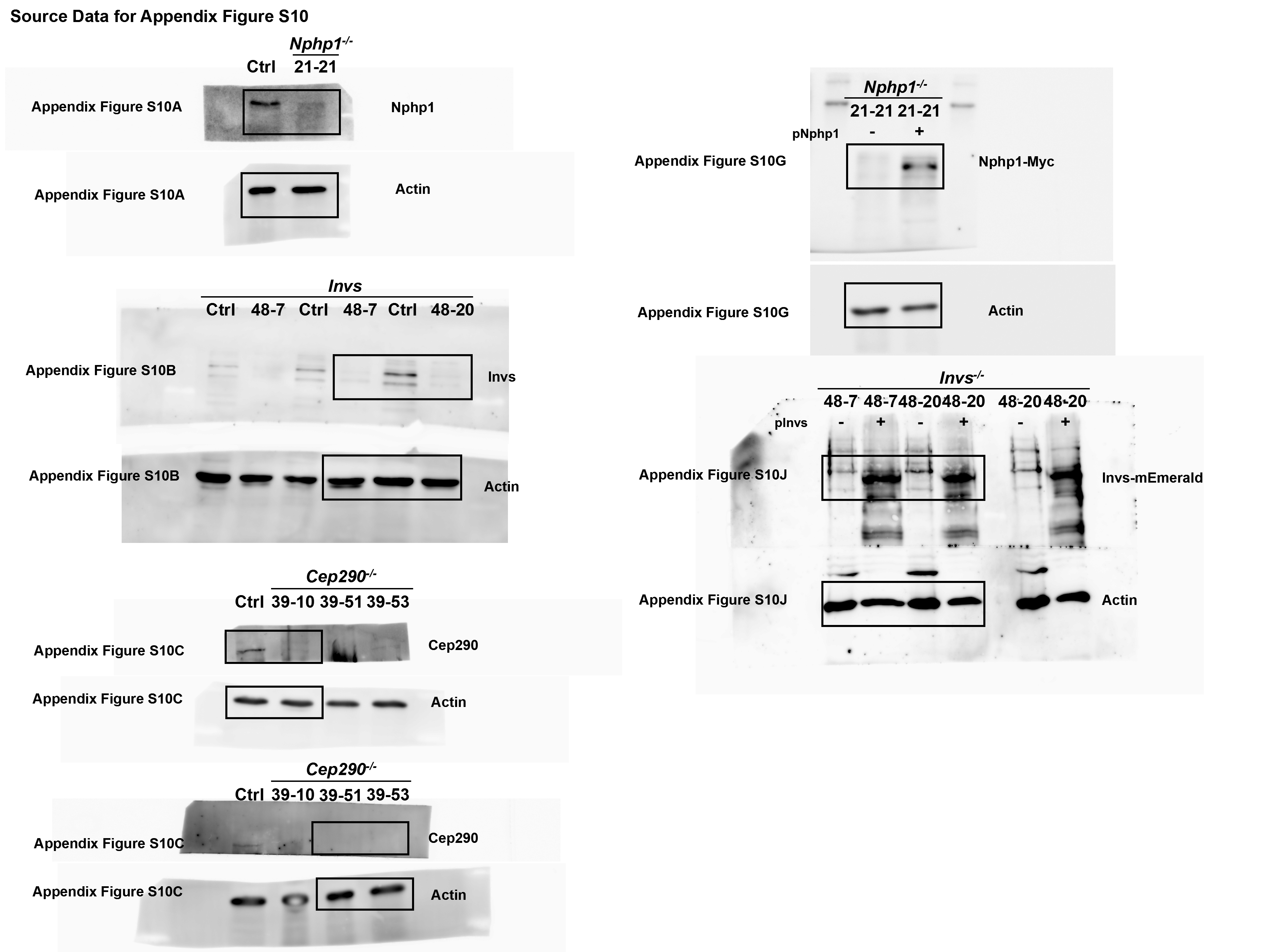

Supplement: Supplementary file 5 — Source Data for Appendix [file EMBJ-37-e97791-s008.zip › EMBOJ-2017-97791R_Appendix_Source_Data/EMBOJ-2017-97791R_Source_Data_for_Appendix_Figure_S10.tif]

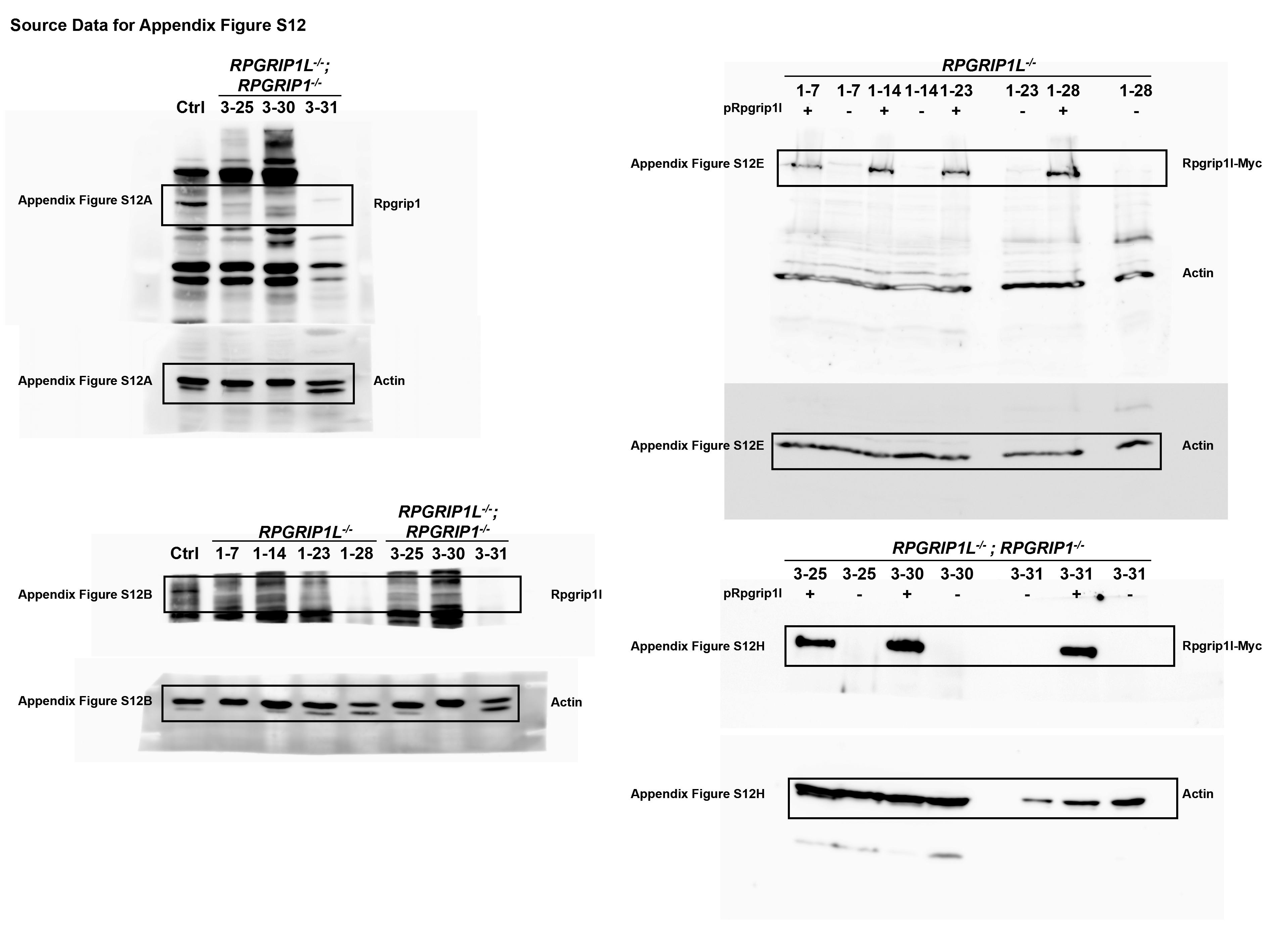

Supplement: Supplementary file 5 — Source Data for Appendix [file EMBJ-37-e97791-s008.zip › EMBOJ-2017-97791R_Appendix_Source_Data/EMBOJ-2017-97791R_Source_Data_for_Appendix_Figure_S12.tif]

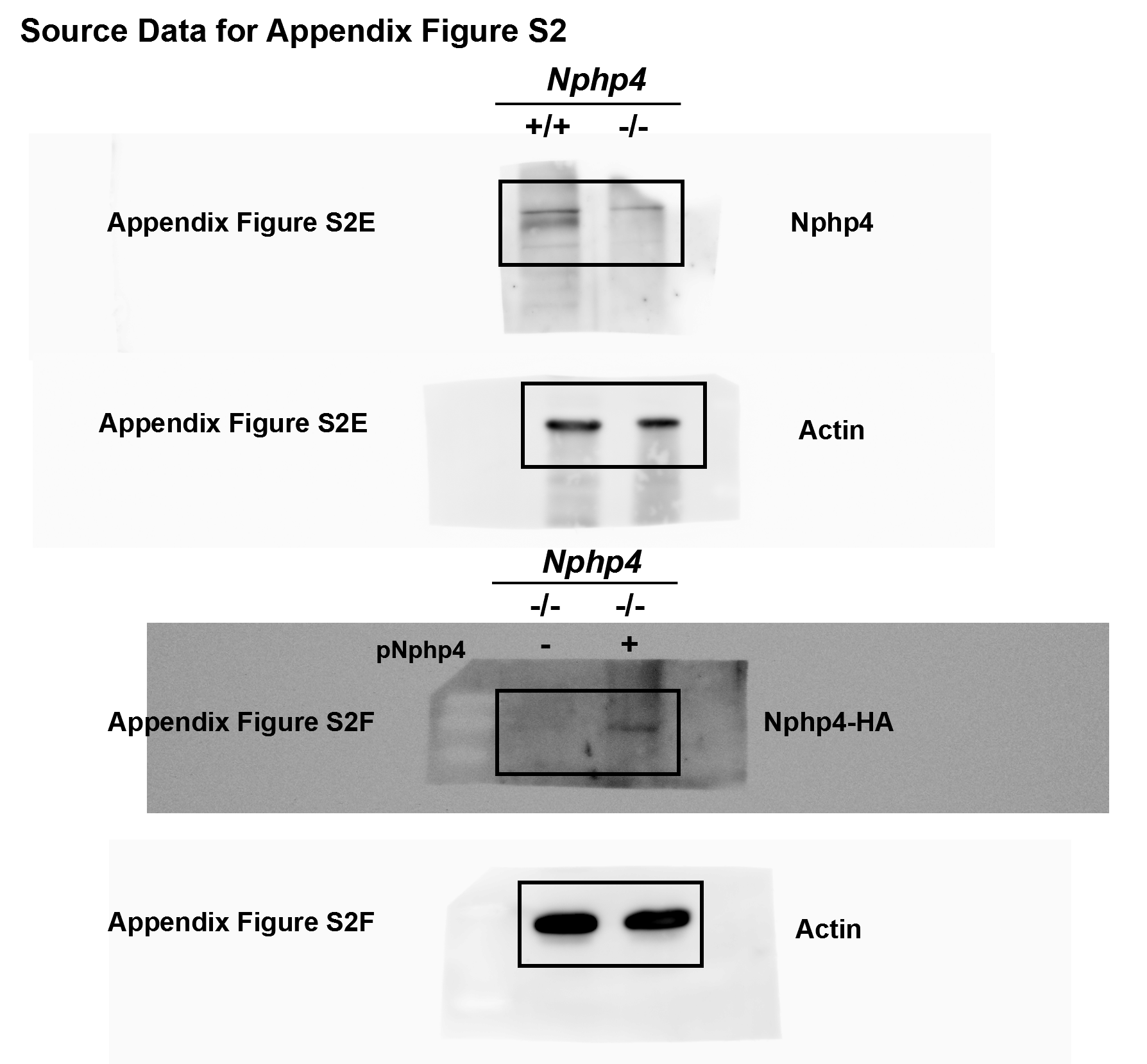

Supplement: Supplementary file 5 — Source Data for Appendix [file EMBJ-37-e97791-s008.zip › EMBOJ-2017-97791R_Appendix_Source_Data/EMBOJ-2017-97791R_Source_Data_for_Appendix_Figure_S2.tif]

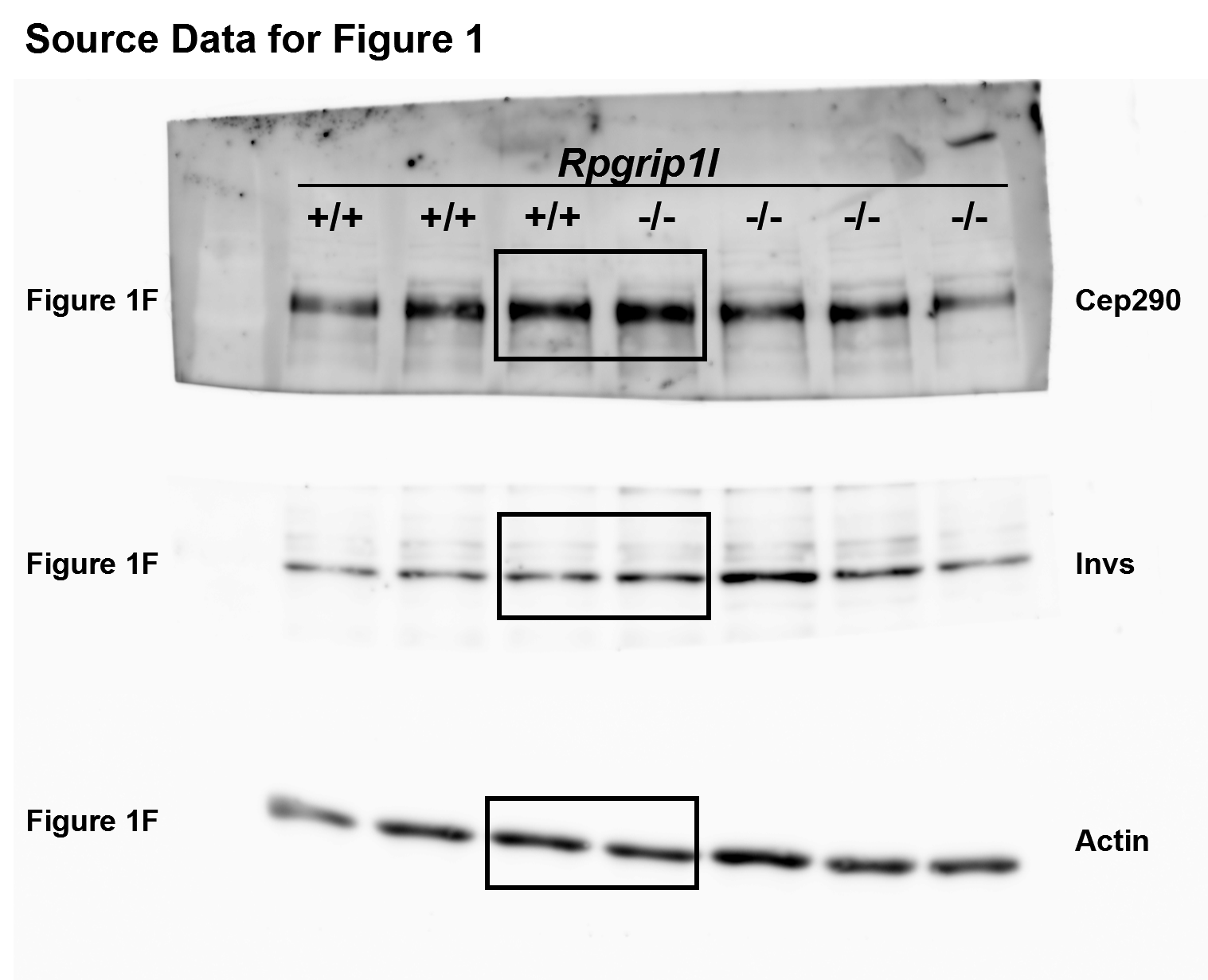

Supplement: Supplementary file 7 — Source Data for Figure 1 [file EMBJ-37-e97791-s005.tif]

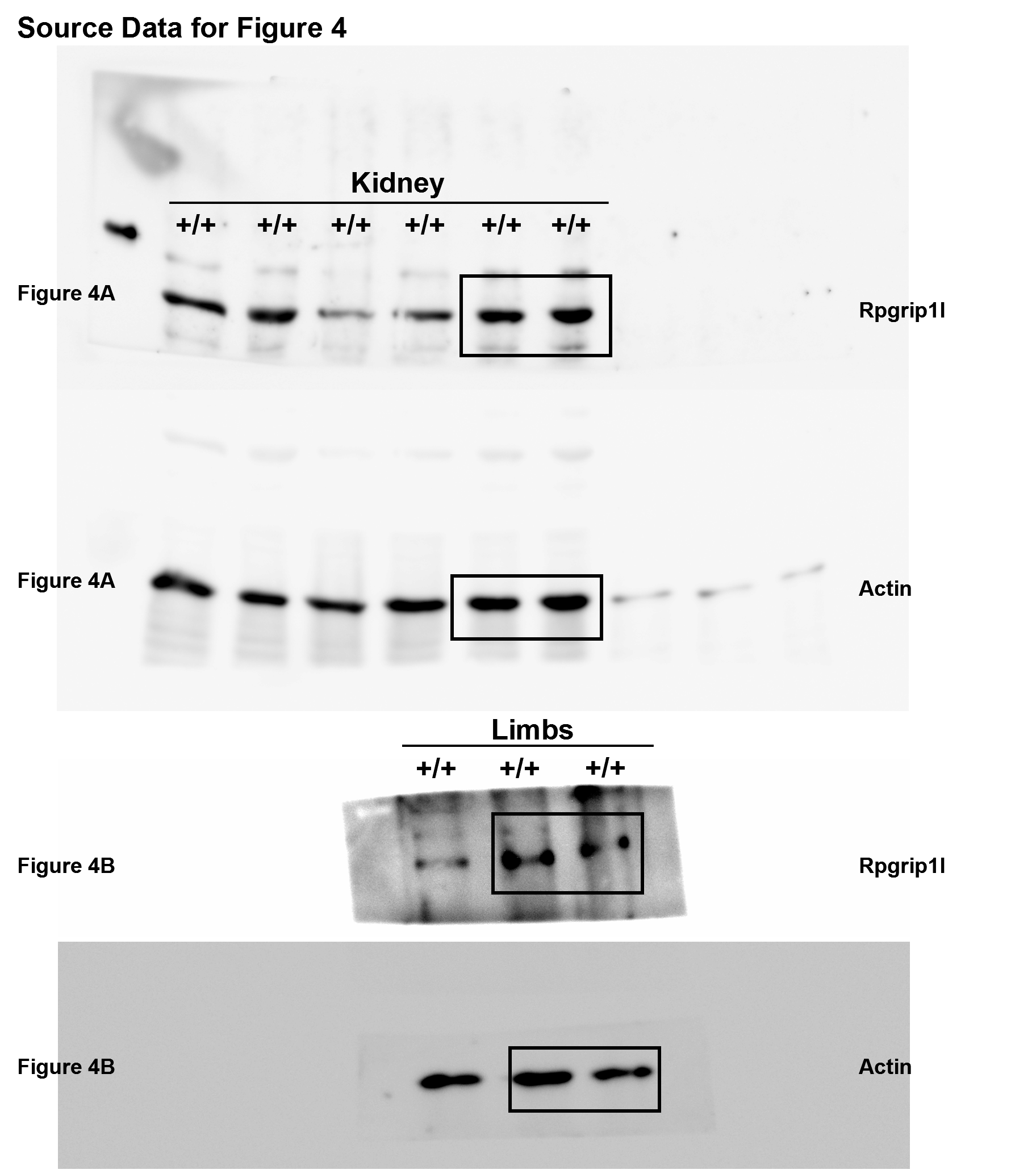

Supplement: Supplementary file 8 — Source Data for Figure 4 [file EMBJ-37-e97791-s006.tif]

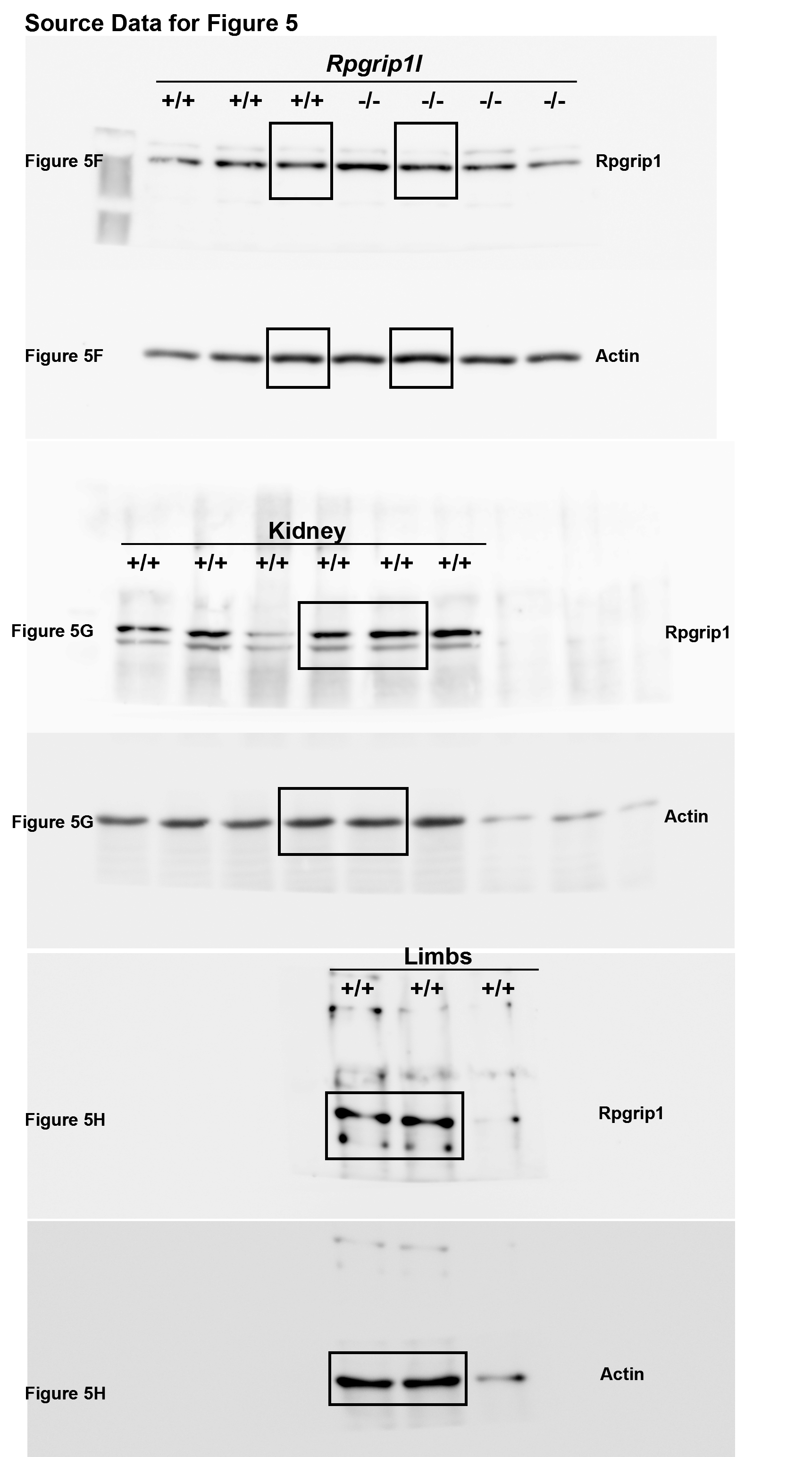

Supplement: Supplementary file 9 — Source Data for Figure 5 [file EMBJ-37-e97791-s007.tif]
